# Experimental and natural infections of white-tailed sea eagles (*Haliaeetus albicilla*) with high pathogenicity avian influenza virus of H5 subtype

**DOI:** 10.3389/fmicb.2022.1007350

**Published:** 2022-10-03

**Authors:** Yoshikazu Fujimoto, Kohei Ogasawara, Norikazu Isoda, Hitoshi Hatai, Kosuke Okuya, Yukiko Watanabe, Ayato Takada, Yoshihiro Sakoda, Keisuke Saito, Makoto Ozawa

**Affiliations:** ^1^Joint Faculty of Veterinary Medicine, Kagoshima University, Kagoshima, Japan; ^2^Joint Graduate School of Veterinary Medicine, Kagoshima University, Kagoshima, Japan; ^3^Institute for Raptor Biomedicine Japan, Kushiro, Japan; ^4^Laboratory of Microbiology, Department of Disease Control, Faculty of Veterinary Medicine, Hokkaido University, Sapporo, Japan; ^5^International Collaboration Unit, International Institute for Zoonosis Control, Hokkaido University, Sapporo, Japan; ^6^Division of Global Epidemiology, International Institute for Zoonosis Control, Hokkaido University, Sapporo, Japan

**Keywords:** high pathogenicity avian influenza virus, white-tailed sea eagles, H5 subtype, clade 2.3.4.4, viral pathogenicity

## Abstract

White-tailed sea eagle (*Haliaeetus albicilla*), a regionally rare species of raptor, is threatened in several countries. To assess the risk of H5 high pathogenicity avian influenza (HPAI) viral infection in rare bird species, we performed experimental infections with a GS/GD96-lineage H5N6 HPAI virus of clade 2.3.4.4e in white-tailed sea eagles. Additionally, during the winter of 2020–2021 in Japan, we accidentally encountered a white-tailed sea eagle that had a fatal outcome due to natural infection with a GS/GD96-lineage H5N8 HPAI virus of clade 2.3.4.4b, allowing us to compare experimental and natural infections in the same rare raptor species. Our experiments demonstrated the susceptibility of white-tailed sea eagles to the GS/GD96-lineage H5 HPAI virus with efficient replication in systemic organs. The potential for the viruses to spread within the white-tailed sea eagle population through indirect transmission was also confirmed. Comprehensive comparisons of both viral distribution and histopathological observations between experimentally and naturally infected white-tailed sea eagles imply that viral replication in the brain is responsible for the disease severity and mortality in this species. These findings provide novel insights into the risk assessment of H5 HPAI viral infection in white-tailed sea eagles, proper diagnostic procedures, potential risks to artificially fed eagle populations and persons handling superficially healthy eagles, potential impact of intragastric infection on eagle outcomes, and possibility of severity of the disease being attributed to viral replication in the brain.

## Introduction

Since the high pathogenicity avian influenza (HPAI) virus of the H5 subtype, A/Goose/Guangdong/1/96 (H5N1) (GS/GD96), was initially isolated from a goose belonging to a farm in Guangdong Province, China in 1996, the GS/GD96-lineage H5 HPAI viruses have spread in many countries in East Asia, Southeast Asia, Europe, and Africa Xu et al., 1999; Smith et al., 2015. Furthermore, the GS/GD96-lineage virus spread from Asia to North America in 2014, most likely owing to the long-distance migration of wild birds (Global Consortium for H5N8 Related Influenza Viruses, 2016). Through the worldwide spread of GS/GD96-lineage viruses, hemagglutinin (HA) genes have evolved continuously and rapidly and are now divided into 10 distinct clades with multiple subclades (WHO/OIE/FAO H5N1 Evolution Working Group, 2008). In addition to waterfowl, which are the primary natural reservoir for avian influenza viruses, a wide variety of wild terrestrial birds and mammalian species have been confirmed to be susceptible to GS/GD96-lineage of H5 HPAI viruses in both experimental and natural infection cases (Kaplan and Webby, 2013; El-Shesheny et al., 2020; Li et al., 2021).

A recent study (McClure et al., 2018) reported that 52% of 557 raptor species (the orders *Accipitriformes* and *Falconiformes*) had exhibited declining global populations since 1988, and 18% are threatened with extinction. Raptors represent the apex of their food webs, and thus, they have a potential risk of exposure to pathogens, including HPAI viruses, present in their prey. Cases of natural infection of raptors with HPAI viruses have not been frequently explored. For example, among 624 dead birds belonging to nine raptor species found in Germany during the H5N1 HPAI outbreak in 2006, the H5N1 HPAI virus infections were confirmed only in two species: common buzzard (12 positive/385 tested, 3.1%) and peregrine falcon (2 positive/6 tested, 33.3%) (van den Brand et al., 2015). However, since H5 HPAI viruses of clade 2.3.4.4 became widespread in 2016 (European Food Safety Authority et al., 2018), more raptor species have suffered from natural infections with H5 HPAI viruses. To date, H5 HPAI viruses of clade 2.3.4.4 have been detected in more than 15 raptor species (Van Borm et al., 2005; Couacy-Hymann et al., 2009; Khan et al., 2009; Shivakoti et al., 2010; Hall et al., 2015; van den Brand et al., 2015; Hiono et al., 2017; Okamatsu et al., 2017; European Food Safety Authority et al., 2018; Globig et al., 2018; Krone et al., 2018; Shearn-Bochsler et al., 2019; Isoda et al., 2020; Liang et al., 2021; Verhagen et al., 2021). Therefore, H5 HPAI viruses, especially those in clade 2.3.4.4, are deemed to be an emerging and serious risk factor for threatened raptor species.

White-tailed sea eagles (*Haliaeetus albicilla*) are widely distributed, ranging from the Netherlands in Western Europe to Japan in the Far East. In Europe, since a dramatic decrease in its population between 1950 and 1970, many species-specific monitoring and conservation activities have been conducted, resulting in population growth in recent years, with ~70% of the global population (24,200–49,000 birds) residing in European countries (https://danubeparks.tabardi.hr/sharepoint/public/1589807510_uploads.pdf). Currently, this precious bird species for nature protection in Europe is listed as the least concerning one in the International Union for Conservation of Nature Red List of Threatened Species (https://www.iucn.org/). In contrast to the success of European protection policies, white-tailed sea eagles are still considered to be a more threatened bird species in several countries. In Japan, where the population size is relatively small (700–900 birds), the white-tailed sea eagle is categorized as a vulnerable species in the Japanese Red List 2020 (Ministry of the Environment, Government of Japan) and is defined as a national rare wild animal and plant species (Ministry of the Environment), a natural monument (Agency for Cultural Affairs, Government of Japan), and partly exploited as a tourism resource. The recent global spread of H5 HPAI viruses can affect populations of white-tailed sea eagles. In fact, intermittent outbreaks of H5 HPAI viruses of clade 2.3.4.4b in Europe have resulted in several infection cases of white-tailed sea eagles (van den Brand et al., 2015; European Food Safety Authority et al., 2018; Krone et al., 2018). For improved conservation of the white-tailed sea eagle, the risk of H5 HPAI virus infection needs to be assessed.

For risk assessment of H5 HPAI virus infection in certain animal species, experimental infections in the animal species would provide critical information, including the susceptibility of hosts, viral transmissibility, and organ tropism. To date, two raptor species were subjected to experimental infections with the GS/GD96-lineage H5 HPAI viruses of clade 2.2: Gyr-Saker (*Falco rusticolus* × *Falco cherrug*) hybrid falcon (Lierz et al., 2007; Bertran et al., 2012) and American kestrel (*Falco sparverius*) (Hall et al., 2009). In this study, we assessed experimental infections of white-tailed sea eagles with a GS/GD96-lineage H5N6 HPAI virus of clade 2.3.4.4e, as approved by the concerned authorities. Additionally, during the winter of 2020–2021 in Japan, we accidentally encountered a white-tailed sea eagle that had a fatal outcome due to natural infection with a GS/GD96-lineage H5N8 HPAI virus of clade 2.3.4.4b, allowing us to compare experimental and natural infections in the same rare raptor species.

## Materials and methods

### Ethics statement

Three white-tailed sea eagles were included in this study. Adult (over 10 years old) female, subadult (~2 years old) female, and adult (over 10 years old) male white-tailed sea eagles that had been rescued and reared at the Institute for Raptor Biomedicine Japan (Kushiro, Japan) under the protection propagation program (Ministry of the Environment) were selected for this study. All three white-tailed sea eagles were involved in individual traffic accidents and underwent surgery to amputate one of their wings. Thus, they were considered to have no prospects of returning to the wild. The use of these three birds in this study was approved by the Ministry of Environment and Agency for Cultural Affairs. All animal experiments were conducted at Hokkaido University (Sapporo, Japan) and were approved by the Institutional Animal Care and Use Committee of Hokkaido University (permission number: 19-0157).

### Experimental infection with H5N6 HPAI virus

The three white-tailed sea eagles were transported from the Institute for Raptor Biomedicine Japan (Kushiro) to Hokkaido University (Sapporo), ~250 km from the institute. These eagles were kept individually in stout plastic cages located in negatively pressured isolation rooms with biosafety level 3 facility, at the International Institute for Zoonosis Control, Hokkaido University. They were allowed to acclimate for 3 days prior to experimental inoculation. Three Okhotsk atka mackerel (*Pleurogrammus azonus*) were fed to each bird daily. The absence of specific antibodies against the H5 HPAI virus in the sera of white-tailed sea eagles was confirmed by a neutralization test performed using cultured cells and A/black swan/Akita/1/2016 (H5N6) prior to experimental infection, following a previous report (Fujimoto et al., 2019). The body temperature and weight of the eagles were measured using a digital hanging scale and a rectal thermometer, respectively, immediately before viral inoculation and every morning after viral inoculation.

Two out of the three white-tailed sea eagles (bird A: adult female; bird B: adult male) were intranasally inoculated with 10^7.3^ 50% egg infectious dose (EID_50_) of A/black swan/Akita/1/2016 (H5N6) (Akita/H5N6) belonging to Group E in clade 2.3.4.4 (Isoda et al., 2020). This inoculum dose was equivalent to 1,000-fold 50% chicken lethal dose (Hiono et al., 2017). As we aimed to demonstrate the susceptibility of white-tailed sea eagles to HPAI virus infection, a high inoculum dose was selected in this experimental study. Akita/H5N6 strain was selected to inoculate the eagles experimentally because H5N6 HPAI viruses that are genetically close to the Akita/H5N6 strain were isolated from several dead raptors, including peregrine falcons (*Falco peregrinus*), goshawks (*Accipiter gentilis*), and gray-faced buzzards (*Buteo japonicus*) in northern Japan during the winter of 2016–2017. Birds A and B were monitored for 3 and 8 days, respectively, for the development of clinical signs. To examine whether the H5N6 HPAI virus could spread among white-tailed sea eagles without direct contact, the remaining naïve white-tailed sea eagle (bird C: subadult female) was housed in a cage placed ~30 cm away from the inoculated bird B. The lower parts of the cages consisted of lattice architecture, allowing the indirect transmission of the viruses between birds B and C. Bird C was monitored for clinical signs up to day 7 of the experiment. Although the distance between the cages of birds A and C was over 2 m, all cages, including those of bird B, were placed in the same experimental room. To demonstrate viral replication in organs on different days after infection, we euthanized birds A and B on days 3 and 8 post-inoculation and collected their organs. Because bird C was used for an additional experiment, it was not euthanized on day 7 post-inoculation; thus, viral titers from its organs were not available.

Conjunctival, oropharyngeal, and cloacal swabs and body feathers were collected daily in 3 mL viral transport medium. One milliliter of blood sample was collected using BD vacutainer plastic whole blood tube K2EDTA (Becton Dickinson and Company, Franklin Lakes, NJ) on days 3 and 6 post-inoculation. Virus recovery from swabs and blood samples was performed on 10-day-old embryonated chicken eggs, as described previously (Shivakoti et al., 2010). The detection limit was below 0.5 log EID_50_/mL. To investigate viral replication and histopathological change in organs from the inoculated eagles, birds A and B were euthanized by intravenous injection of sodium pentobarbital on days 3 and 8 post-inoculation, respectively. After sacrifice, organs including the brain, lung, heart, liver, proventriculus, pancreas, spleen, colon, and kidney of infected birds were collected, and viral titers were determined using the EID_50_ assay with 3 embryonated chicken eggs as described previously (Fujimoto et al., 2010). Although the standard protocols by the OIE recommended the use of five eggs for viral titration, we previously confirmed that there was no significant difference in the resultant virus titers between the use of three and five eggs for viral titration (data not shown). The detection limit was below 1.5 log EID_50_/g. Because bird C was used in an additional experiment, it was not euthanized 7 days post-indirect contact; therefore, viral titer from this bird was not obtained.

### Serum biochemical examination

The collected blood samples were centrifuged at 3,000 rpm at 4°C for 10 min, and 200 μL of the serum was separated from the blood cells for serum biochemical examination. Serum biochemical examinations were performed using the Zoetis Abaxis VetScan VS2 (Abaxis, Union City, CA, USA) with a VetScan Avian/Reptilian Profile Plus Rotor. The test parameters were albumin, aspartate aminotransferase, creatine kinase, glucose, phosphorus, total protein, and uric acid levels.

### Histopathology and immunohistochemistry

The organs collected during necropsy were fixed in 10% neutral buffered formalin, routinely processed, and embedded in paraffin wax. Sections of the organs (3 μm) were stained with hematoxylin and eosin. Labeled polymer immunohistochemical analysis was performed using Histofine MAX PO (Nichirei Biosciences, Tokyo, Japan) with a mouse monoclonal antibody specific for influenza A virus M1 protein as the primary antibody (1 in 1,000 dilution; clone GA2B; Bio-Rad Laboratories, Hercules, CA).

### Natural infection with H5N8 HPAI virus

A debilitated adult white-tailed sea eagle was found in a residential area in Asahikawa, Japan on January 26, 2021. We captured this eagle the next day, in accordance with the protection propagation program (Ministry of the Environment), and transported the bird to the Institute for Raptor Biomedicine, Japan for medical evaluation and treatment. This eagle showed neurological clinical signs, including depression and head shake, accompanied by edema in both legs, and died on January 27, 2021. The lead level in its blood, as measured by Leadcare II (Magellan Diagnostics, North Billerica, MA), was below the detection limit (<3.3 μg/dL), indicating that chance of lead poisoning causing the symptoms could be ruled out. To conduct a screening test for avian influenza, a rapid reverse transcription (RT)-polymerase chain reaction (PCR) was performed using Cobas Influenza A/B & RSV (Roche Diagnostics, Basel, Switzerland). All conjunctival, oropharyngeal, and cloacal swab specimens collected from the dead bird tested positive for the influenza A virus.

We then collected the organs for virus isolation, virus titration, and histopathological examination, as described above. The genome sequence of the isolated virus was determined as previously described (Isoda et al., 2020). The sequencing data were submitted to the NCBI GenBank database and were listed under accession No. MZ235331 to MZ235338. The nucleotide sequences with the coding regions of PB2, PB1, PA, HA, NP, M, and NS gene segments were aligned with their counterparts from representative H5 HPAI viruses retrieved from the GISAID database (http://platform.gisaid.org/) using MUSCLE (Edgar, 2004), and those of the NA gene segment were from representative N8 low-pathogenicity avian influenza viruses. The phylogenetic trees for each viral gene were constructed using MEGA 7 software (https://www.megasoftware.net/) by the maximum likelihood method using 1,000 bootstrap replicates to determine statistical significance. Bootstrap values of >70% are shown at the nodes. The scale bars indicate the number of nucleotide substitutions per site.

## Results

### Experimental infections of white-tailed sea eagles with H5N6 HPAI virus

**Figure 1:**
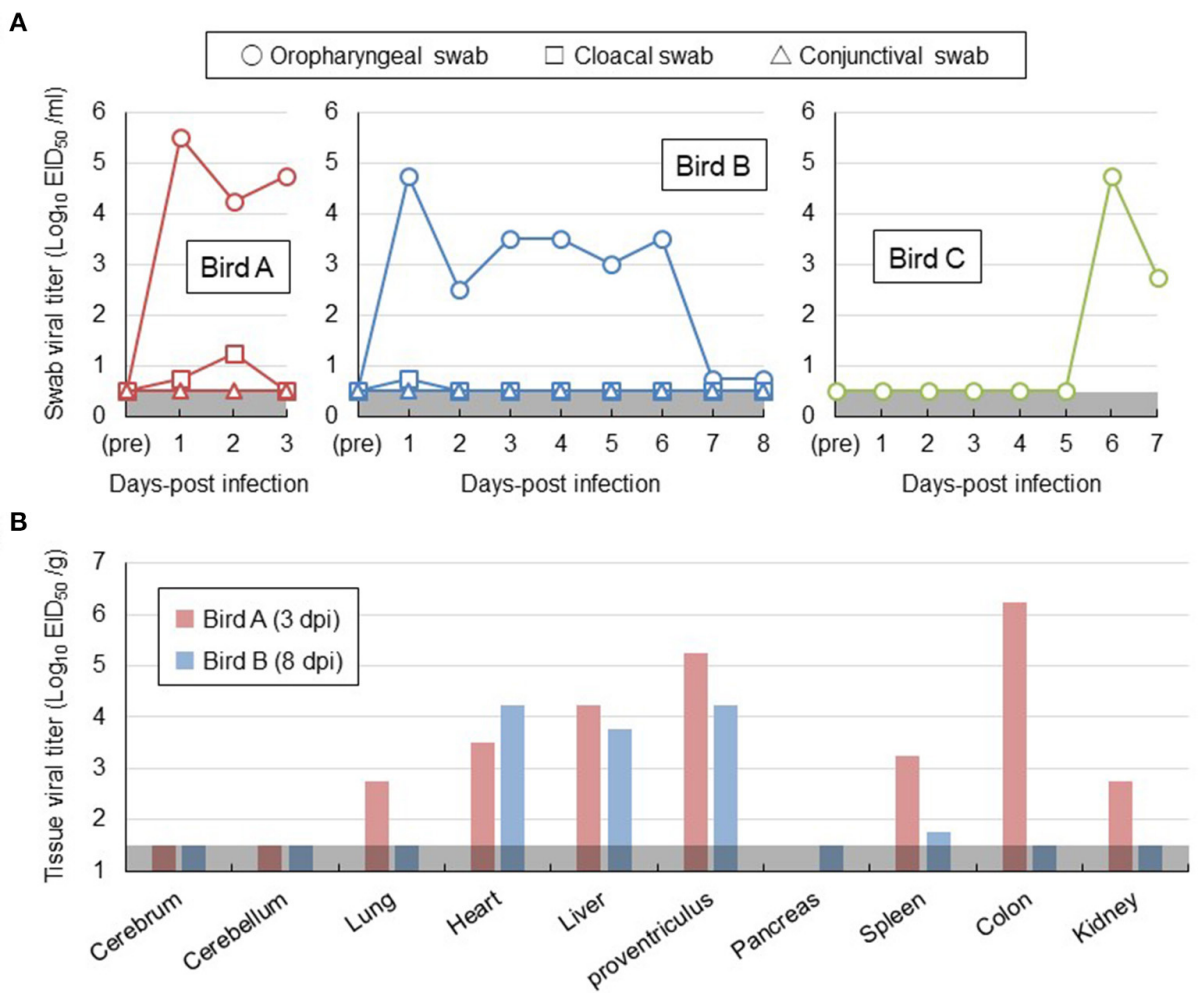
Viral replication in white-tailed sea eagles experimentally infected with H5N6 HPAI virus. **(A)** Temporal change of viral titers in swab samples. Viral titers in oropharyngeal swab (circle), cloacal swab (square), and conjunctival swab (triangle) samples collected from the two inoculated eagles (birds A and B) and from the contact eagle (bird C) that was housed adjacent to bird B were determined using embryonated chicken eggs. The detection limit was below 0.5 log EID_50_/mL. **(B)** Viral titers in organs obtained from the two inoculated eagles. Birds A and B (i.e., the two inoculated eagles) were euthanized on days 3 and 8 post-inoculation, respectively. Organs were collected, homogenized, and subjected to virus titration in embryonated chicken eggs. The detection limit was below 1.5 log EID_50_/g.

For a better understanding of risk management for H5 HPAI viral infection in white-tailed sea eagles, two out of the three eagles (birds A and B) were intranasally inoculated with Akita/H5N6 strain. The remaining eagle (bird C) was placed in a cage adjacent to the inoculated bird B. None of the three eagles exhibited any clinical signs during the experimental period. No marked changes in the body temperature or weight were observed (Supplementary Figures S1A,B). No virus was detected in the conjunctival swabs, feathers, or blood samples of all eagles tested by virus isolation. Nevertheless, oropharyngeal swabs collected from bird A on days 1–3 post-inoculation and bird B on days 1–6 post-inoculation contained viruses, with titers greater than ~10^3.0^ EID_50_/mL (Figure 1A). We cannot rule out the possibility that the peak virus titers in oropharyngeal swabs of birds A and B on day 1 post-inoculation detected a portion of the viral inoculum. In cloacal swabs of birds A and B, a small amount of virus was detected within 2 days post-inoculation (Figure 1A). Moreover, after euthanizing birds A and B on days 3 and 8 post-inoculation, respectively, viruses at titers of 10^1.8^-10^6.2^ EID_50_/g were recovered from all organs examined, except the brain and pancreas (Figure 1B). However, no prominent lesions and viral antigens were found in their major organs upon histopathological examination. These results indicated that white-tailed sea eagles are susceptible to the Akita/H5N6 strain and allow viral replication in various organs. Intriguingly, viruses were detected in oropharyngeal swabs collected from bird C within 6–7 days post-contact, indicating that the viruses were indirectly transmitted among white-tailed sea eagles (Figure 1A).

To further characterize H5 HPAI virus infection in white-tailed sea eagles, blood samples collected from the three eagles, obtained once every 3 days, were subjected to serum biochemical examination (Table 1). Based on reference values measured prior to the experimental infection, serum biochemical changes were restricted to aspartate aminotransferase and creatine kinase, the slightly increased values of which (in birds A and B on day 3 post-inoculation; in bird C on day 6 post-inoculation) were parallel to the status of viral excretion. These changes may be associated with liver and/or kidney damage during the early phases of viral infection. However, no distinct abnormalities were observed in biochemical examination of serum. These results suggest the limited effectiveness of serum biochemical examinations for the diagnosis of HPAI viral infection in white-tailed sea eagles.

**Table 1:** Changes in chemical parameters in blood of the inoculated white-tailed sea eagles.

**Bird ID**	**dpi**	**Chemical parameter concentrations**						
		**AST**	**CK**	**UA**	**GLU**	**PHOS**	**TP**	**ALB**
		**(U/L)**	**(U/L)**	**(mg/dL)**	**(mg/dL)**	**(mg/dL)**	**(g/dL)**	**(g/dL)**
Bird A	0	141	170	2.7	338	2.4	3.6	2.4
	3	328	318	7.4	276	2.6	4.2	2.2
Bird B	0	182	166	5.4	318	2.1	3.8	2.3
	3	238	677	5.1	289	1.0	4.0	2.1
	6	181	384	5.1	304	2.6	4.2	2.4
Bird C	0	164	324	4.2	295	3.0	4.0	2.3
	3	186	368	7.7	299	2.9	4.2	2.4
	6	321	465	2.1	306	1.5	3.7	2.1

AST, aspartate aminotransferase; CK, creatine kinase; UA, uric acid; GLU, glucose; PHOS, phosphorus; TP, total protein; ALB, albumin.

### Natural infection of A white-tailed sea eagle with H5N8 HPAI virus

**Figure 2:**
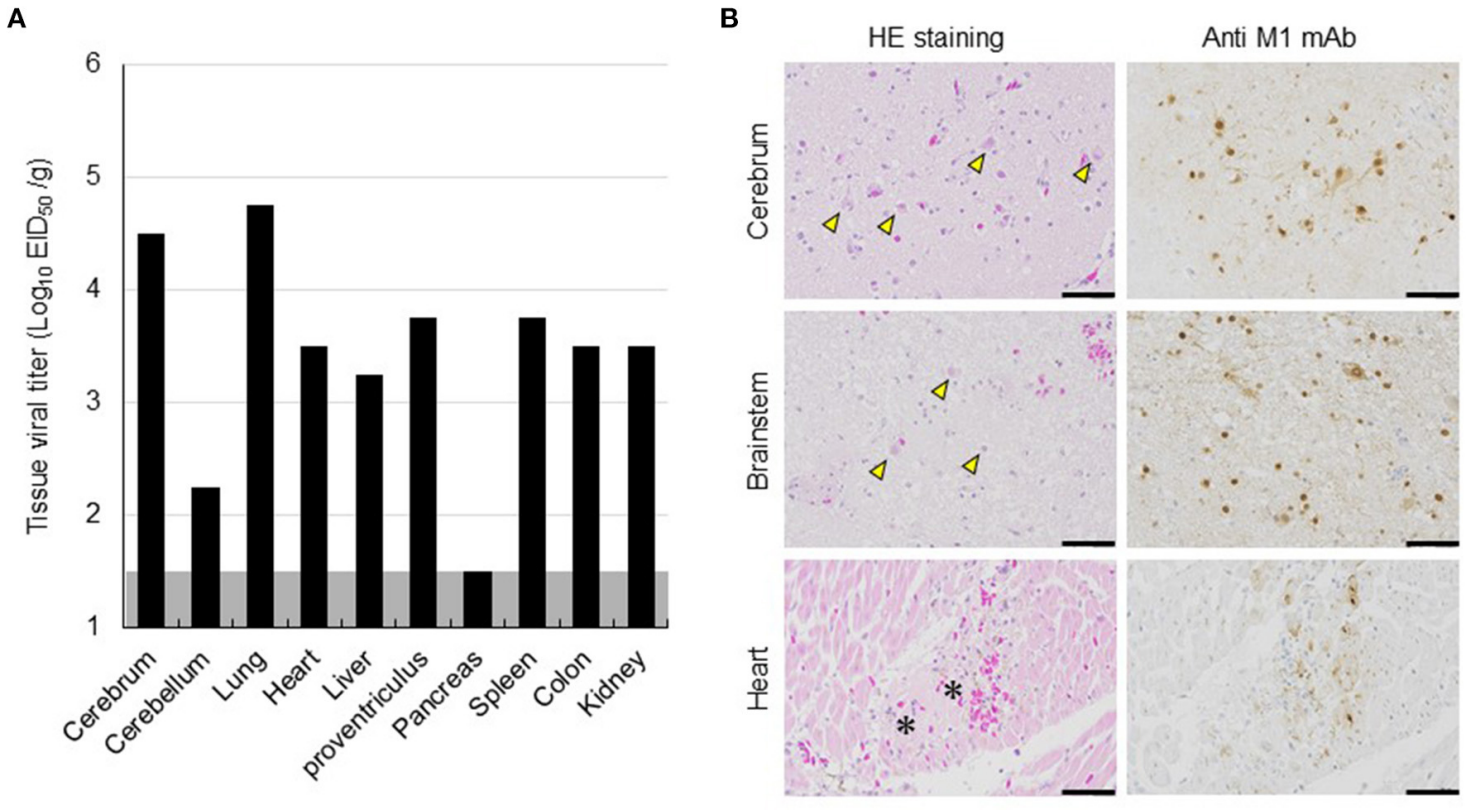
Viral replication and histopathological lesions in organs from a dead white-tailed sea eagle naturally infected with H5N8 HPAI virus. **(A)** Viral titers in organs from the dead white-tailed sea eagle. Organs were collected, homogenized, and subjected to virus titration in embryonated chicken eggs. The detection limit was below 1.5 log EID_50_/g. **(B)** Histopathological findings in the dead white-tailed sea eagle. Sections from the cerebrum, brainstem, and heart of the dead white-tailed sea eagle were stained with hematoxylin and eosin (HE staining) and mouse monoclonal antibody specific for influenza A virus M1 protein (Anti-M1 mAb). Scale bars, 50 μm. Arrowheads and asterisks indicate neuronal necrosis and myocardial degeneration, respectively.

The dead eagle was then transported to Hokkaido University for further diagnoses and dissected at a biosafety level 3 laboratory for viral isolation and pathological examination. The tissue homogenate of the cerebrum was used to inoculate 10-day-old embryonated chicken eggs, and subsequently, influenza A virus A/white-tailed eagle/Hokkaido/20210127001/2021 (H5N8) (Hok/H5N8 strain) was isolated from it. Genetic analyses revealed that the HA gene from the Hok/H5N8 strain belonged to clade 2.3.4.4b. More importantly, a typical virulence-type sequence, REKRRKR/G, was encoded at the HA cleavage site, suggesting the effectiveness of the Hok/H5N8 strain to cause systemic infection in chickens. The virus with titers of 10^2.2^-10^4.8^ EID_50_/g was recovered from most of the organs examined, including the cerebrum and cerebellum (Figure 2A). Microscopically, small foci of myocardial degeneration and necrosis were dispersed throughout the heart (Figure 2B). Multiple neuronal necrosis and nuclear degeneration were observed in the brain sections, and the lesions were broadly expanded in the cerebrum. As observed immunohistochemically, the influenza A viral protein was detected in the degenerated/necrotic myocardial cells in the heart and the neurons in cerebral necrotic areas and brainstem (Figure 2B). No prominent lesions or viral antigens were found in other organs upon histopathological examination. These results indicate that the Hok/H5N8 strain causes systemic and lethal infections in white-tailed sea eagles.

## Discussion

To gain a better understanding of risk management for the GS/GD96-lineage H5 HPAI virus infection in white-tailed sea eagles, one of the rare raptor species, these birds were experimentally infected with the H5N6 HPAI virus of clade 2.3.4.4e, with the approval of the authorities concerned, and compared with a naturally infected eagle. Because detailed conditions such as the transmission route and infectious dose of the virus cannot be controlled in natural infections, the results of the clinical course observed in white-tailed sea eagles in our experimental study may not be applicable to naturally infected cases. In addition, we cannot rule out the possibility that the HA gene subclade difference between Akita/H5N6 (clade 2.3.4.4e) and Hok/H5N8 (clade 2.3.4.4b) strains may be attributed to the diverse clinical symptoms in the infected white-tailed sea eagles. Although none of the three experimentally infected eagles exhibited any clinical signs (Table 1; Supplementary Figure S1), viral shedding from oropharyngeal swabs was detected until 6 days post-inoculation (Figure 1A). Viruses were recovered from various organs, except the cerebrum and cerebellum of two inoculated eagles, until 8 days post-inoculation (Figure 1B). More importantly, viruses were detected in the naïve eagle placed in a cage adjacent to the inoculated eagle at 6–7 days post-contact (Figure 1A), indicating the potential of the viruses for indirect transmission among the white-tailed sea eagle population. Taken together, these findings experimentally confirmed that white-tailed sea eagles are susceptible to the GS/GD96-lineage H5 HPAI viruses of clade 2.3.4.4e that efficiently replicates in systemic organs. Furthermore, a white-tailed sea eagle, which was debilitated with neurological disorders and eventually died, was examined virologically (Figure 2A) and pathologically (Figure 2B). The results revealed that a GS/GD96-lineage H5N8 HPAI virus of clade 2.3.4.4b caused systemic and lethal infections in white-tailed sea eagles.

Krone et al. (2018) reported that HPAI virus-infected white-tailed sea eagles frequently exhibited alert and overexcited behavior. Similarly, the naturally infected white-tailed sea eagle examined in this study showed an extreme response to the human approach (see the Materials and Methods section), with depression and head shake. Notably, these neurological clinical signs were also observed in cases of lead poisoning, which is one of the most significant threats to raptors (Saito, 2009). Rapid and accurate diagnosis is crucial for minimizing the damage caused by HPAI virus infection not only to individual white-tailed sea eagles but also to the surrounding environment. In this study, serum biochemical analyses of experimentally infected white-tailed sea eagles did not reveal abnormalities (Table 1), suggesting low potency of blood biochemical analyses for the diagnosis of HPAI virus infection in white-tailed sea eagles. Among the biological samples that were easily collected from debilitated eagles, only oropharyngeal swabs, at least at the early stages of infection, were considered to be reliable for diagnosis (Figure 1A). Therefore, we propose that viral gene detection in oropharyngeal swabs is the most appropriate diagnostic method for HPAI virus infection in white-tailed sea eagles.

A previous study (Ulrich et al., 2018) reported that histopathological lesions and viral RNAs were detected in the brain and lungs of white-tailed sea eagles infected with H5N8 HPAI virus. In addition, Krone et al. (2018) detected viral antigens only in the brain of an HPAI virus-infected white-tailed sea eagle that had shed viruses in oropharyngeal swabs. However, gross lesions were rarely observed or absent in the organs investigated in these studies. Similarly, although we detected viruses in high titer in the heart, liver, and proventriculus of the inoculated white-tailed sea eagles (Figure 1B), gross lesions or viral antigens were not observed in the organs. These results suggest the possibility that HPAI viral replication sites in organs of white-tailed sea eagles may be restricted, and thus, pathological findings are not consistent with virological data. These findings also imply that HPAI virus infections in white-tailed sea eagles may be overlooked during routine necropsy. One of the most important insights provided by this study is that persons (e.g., veterinarians) handling white-tailed sea eagles and their samples, even when the birds look healthy, may be exposed to a serious risk of infection by HPAI virus. In fact, H5N8 HPAI virus of clade 2.3.4.4b is of concern for public health because the first human cases caused by the viral infection were recorded in December 2020 (Pyankova et al., 2021).

In October 2020, a HPAI virus, A/northern pintail/Hokkaido/M13/2020 (H5N8), was isolated from a fecal sample collected at Lake Komuke, approximately 100 km from the site where the Hok/H5N8 strain-infected white-tailed sea eagle was captured on January 27, 2021. Using genetic analyses and antigenic cartographs, Isoda et al. (2020) demonstrated that the northern pintail isolate belongs to clade 2.3.4.4b. Comparison of sequence data between the northern pintail isolate and Hok/H5N8 strain demonstrated that seven gene segments, including the HA gene, were highly similar (99.2–99.9%), except for the NP gene segment (96.3%) (Supplementary Figure S2). Moreover, during the winter of 2021–2022, at least 15 debilitated or dead white-tailed sea eagles (as of April 30, 2022), and other raptor species including northern goshawk (*Accipiter gentilis*), mountain hawk-eagle (*Nisaetus nipalensis*), and black kite (*Milvus migrans*) found in Hokkaido, were confirmed to be infected by H5 HPAI viruses. Notably, H5 HPAI viruses have also been detected in hundreds of dead jungle crows (*Corvus macrorhynchos*) in Hokkaido during the same period. These results suggest that one of the major sources of infection in white-tailed sea eagles is their infected prey, such as migratory birds that act as natural reservoirs of viruses and/or accidentally infected resident birds. Nevertheless, our experimental results indicate that infected white-tailed sea eagles may also play a role as sources of infection, even *via* indirect transmission (Figure 1A). According to the tourism resources, some of the wild white-tailed sea eagles in Hokkaido are artificially fed, resulting in unusually dense populations. But as these artificial situations likely increase the risk of virus transmission among birds, we propose that artificial feeding of wild white-tailed sea eagles should be firmly restricted.

The different clinical consequences in white-tailed sea eagles experimentally and naturally infected with HPAI virus are potentially associated with several factors, including pathogenicity of viral strain, field-specific secondary factors, duration of infection, and virus invasion route. Although there have been no reports on the experimental comparison between the H5 HPAI viruses of clades 2.3.4.4b and 2.3.4.4e, with respect to their pathogenicity in chickens, it is possible that the Hok/H5N8 strain is more pathogenic in white-tailed sea eagles than the Akita/H5N6 strain. The naturally infected eagle lived in a dirtier environment than the experimentally infected counterparts, and thus, the former had a higher possibility of being infected with other pathogens and/or exposed to hazardous substances. While we had time constraints (only for 8 days post-inoculation) for monitoring the experimentally infected eagles, the date of infection for the natural infection case could not be specified. Although viral shedding in swabs gradually decreased during the experimental period (Figure 1A), H5 HPAI viruses may require a longer time to cause symptomatic infection in white-tailed sea eagles. The route of the natural inoculation might be responsible for the death of the naturally infected eagle. Although the route could not be identified, wild eagles were most likely infected intragastrically *via* infected prey that includes migratory ducks. In fact, intragastrical inocula of H5N1 HPAI viruses have been experimentally demonstrated to cause systemic and lethal infections in ferrets and hamsters (Shinya et al., 2011). In contrast, Bertran et al. (2012) demonstrated that there were similarities in HPAI viral infection dynamics, including clinical onset, viral shedding, and mortality, between falcons inoculated through the viral intranasal route and those that were fed virus-infected chickens. Importantly, the contradictory findings between experimental and natural infections imply the possibility of H5 HPAI virus shedding from asymptomatic white-tailed sea eagles. High level of viral replication in the brains of dead raptors, infected either experimentally or naturally, were common (Lierz et al., 2007; Shivakoti et al., 2010; Bertran et al., 2012; Hall et al., 2015; Krone et al., 2018; Shearn-Bochsler et al., 2019). In accordance with this finding, a high viral titer of 10^4.5^ EID_50_/g and multiple viral antigens were detected in the cerebra of the naturally infected white-tailed sea eagle (Figure 2) but not in those of experimentally infected eagles. It suggests that viral replication in the brain is responsible for disease severity and mortality in white-tailed sea eagles.

In conclusion, we demonstrated, through experimental viral infection, the susceptibility of white-tailed sea eagles to the GS/GD96-lineage H5 HPAI virus, which efficiently replicated in systemic organs. The potency of the viruses to spread among the white-tailed sea eagle population through indirect transmission was also confirmed. Comprehensive comparisons of viral distribution and histopathological analyses between experimentally and naturally infected white-tailed sea eagles imply that viral replication in the brain is responsible for disease severity and mortality in this species. Because the number of white-tailed sea eagles used in this study was small, care should be taken in generalizing these findings. However, our findings provide novel insights into the risk assessment of H5 HPAI virus infection in white-tailed sea eagles, including proper diagnostic procedures, artificial feeding creating potential risks for eagle populations, potential risk of viral exposure while handling seemingly healthy eagles, potential impact of intragastric infection on eagle outcomes, and possible effectiveness of viral replication in the brain for the severity of disease.

## Data availability statement

The datasets presented in this study can be found in online repositories. The names of the repository/repositories and accession number(s) can be found in the article/Supplementary material.

## Ethics statement

The animal study was reviewed and approved by the Institutional Animal Care and Use Committee of Hokkaido University.

## Author contributions

YF, KOg, YS, KS, and MO designed research. YF, KOg, NI, HH, KOk, YW, AT, YS, KS, and MO performed research. YF, KOg, HH, and MO analyzed data. YS and MO acquired funding. YF, KOg, and MO wrote the paper. All authors contributed to the article and approved the submitted version.

## Funding

This study was supported by Environment Research and Technology Development (grant numbers JPMEERF18S20103 and JPMEERF18S20104).

## Conflict of interest

The authors declare that the research was conducted in the absence of any commercial or financial relationships that could be construed as a potential conflict of interest.

## Publisher's note

All claims expressed in this article are solely those of the authors and do not necessarily represent those of their affiliated organizations, or those of the publisher, the editors and the reviewers. Any product that may be evaluated in this article, or claim that may be made by its manufacturer, is not guaranteed or endorsed by the publisher.
