# Supplementary material for: Experimental and natural infections of white-tailed sea eagles (Haliaeetus albicilla) with high pathogenicity avian influenza virus of H5 subtype
Source: Front Microbiol. 2022 Oct 3;13:1007350. doi: 10.3389/fmicb.2022.1007350 (PMC9574225; doi:10.3389/fmicb.2022.1007350)
Supplement: Supplementary file 1 [file Presentation_1.PPTX]

## Slide 1
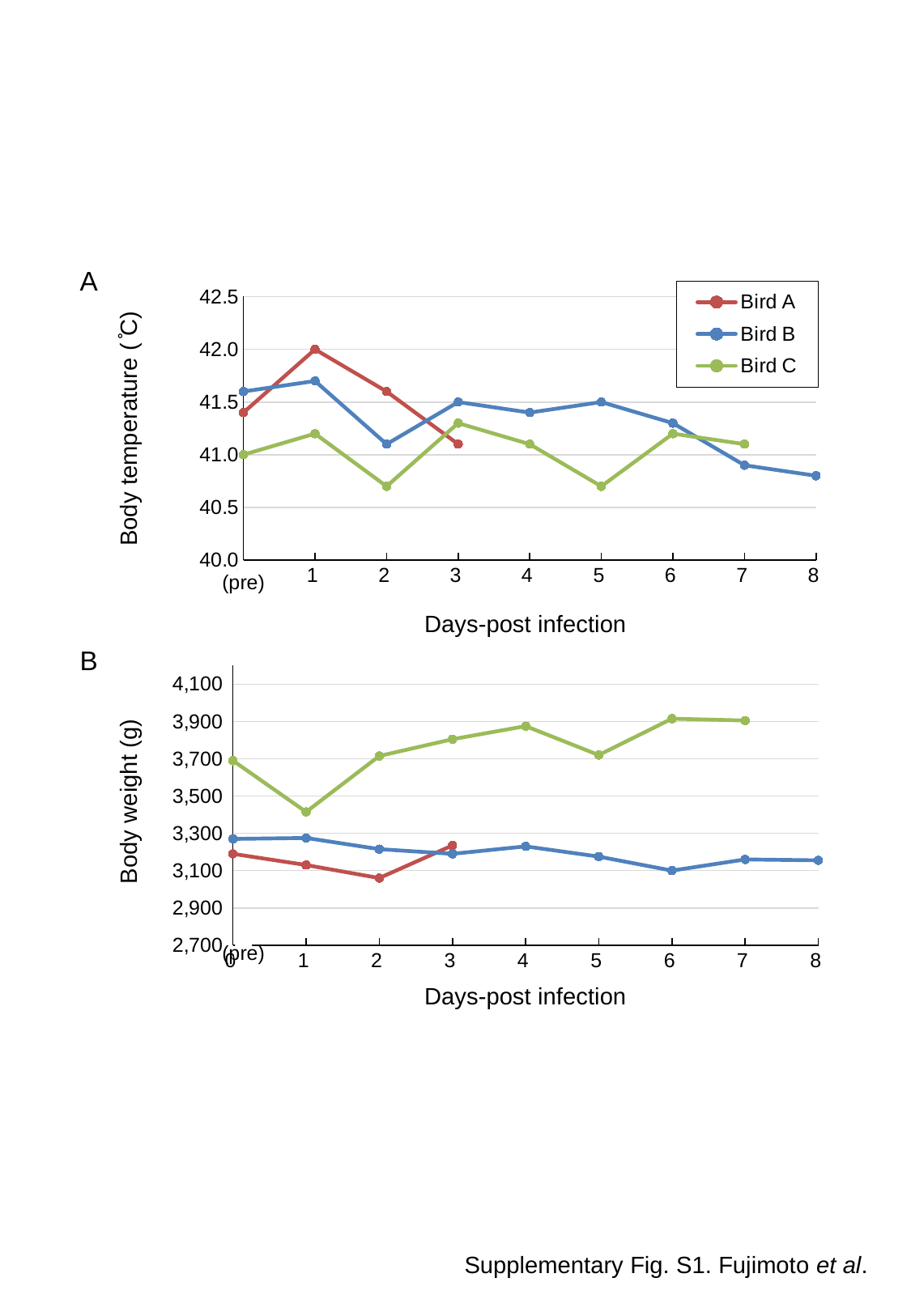

A
### Chart
| Category | | | |
|---|---|---|---|
| 0 | 41.4 | 41.6 | 41.0 |
| 1 | 42.0 | 41.7 | 41.2 |
| 2 | 41.6 | 41.1 | 40.7 |
| 3 | 41.1 | 41.5 | 41.3 |
| 4 | None | 41.4 | 41.1 |
| 5 | None | 41.5 | 40.7 |
| 6 | None | 41.3 | 41.2 |
| 7 | None | 40.9 | 41.1 |
| 8 | None | 40.8 | None |Body temperature ( ̊C)
Days-post infection
B
### Chart
| Category | | | |
|---|---|---|---|
| 0 | 3190.0 | 3270.0 | 3690.0 |
| 1 | 3130.0 | 3275.0 | 3415.0 |
| 2 | 3060.0 | 3215.0 | 3715.0 |
| 3 | 3235.0 | 3190.0 | 3805.0 |
| 4 | None | 3230.0 | 3875.0 |
| 5 | None | 3175.0 | 3720.0 |
| 6 | None | 3100.0 | 3915.0 |
| 7 | None | 3160.0 | 3905.0 |
| 8 | None | 3155.0 | None |Body weight (g)
Days-post infection
(pre)
(pre)
Supplementary Fig. S1. Fujimoto et al.

## Slide 2
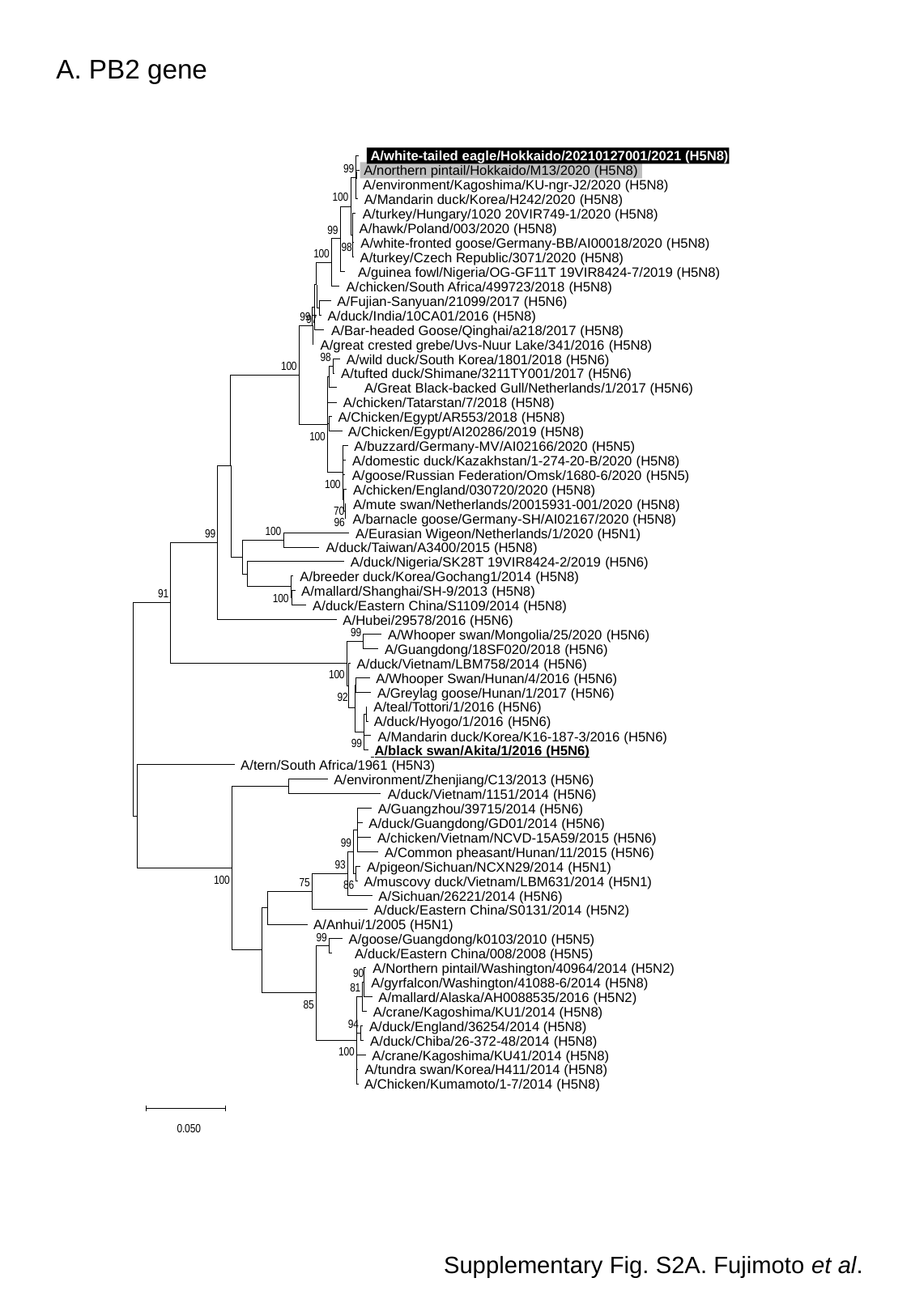

A. PB2 gene
 A/white-tailed eagle/Hokkaido/20210127001/2021 (H5N8)
 A/northern pintail/Hokkaido/M13/2020 (H5N8)
 A/environment/Kagoshima/KU-ngr-J2/2020 (H5N8)
 A/Mandarin duck/Korea/H242/2020 (H5N8)
 A/turkey/Hungary/1020 20VIR749-1/2020 (H5N8)
 A/hawk/Poland/003/2020 (H5N8)
 A/white-fronted goose/Germany-BB/AI00018/2020 (H5N8)
 A/turkey/Czech Republic/3071/2020 (H5N8)
 A/guinea fowl/Nigeria/OG-GF11T 19VIR8424-7/2019 (H5N8)
 A/chicken/South Africa/499723/2018 (H5N8)
 A/Fujian-Sanyuan/21099/2017 (H5N6)
 A/duck/India/10CA01/2016 (H5N8)
 A/Bar-headed Goose/Qinghai/a218/2017 (H5N8)
 A/great crested grebe/Uvs-Nuur Lake/341/2016 (H5N8)
 A/wild duck/South Korea/1801/2018 (H5N6)
 A/tufted duck/Shimane/3211TY001/2017 (H5N6)
 A/Great Black-backed Gull/Netherlands/1/2017 (H5N6)
 A/chicken/Tatarstan/7/2018 (H5N8)
 A/Chicken/Egypt/AR553/2018 (H5N8)
 A/Chicken/Egypt/AI20286/2019 (H5N8)
 A/buzzard/Germany-MV/AI02166/2020 (H5N5)
 A/domestic duck/Kazakhstan/1-274-20-B/2020 (H5N8)
 A/goose/Russian Federation/Omsk/1680-6/2020 (H5N5)
 A/chicken/England/030720/2020 (H5N8)
 A/mute swan/Netherlands/20015931-001/2020 (H5N8)
 A/barnacle goose/Germany-SH/AI02167/2020 (H5N8)
 A/Eurasian Wigeon/Netherlands/1/2020 (H5N1)
 A/duck/Taiwan/A3400/2015 (H5N8)
 A/duck/Nigeria/SK28T 19VIR8424-2/2019 (H5N6)
 A/breeder duck/Korea/Gochang1/2014 (H5N8)
 A/mallard/Shanghai/SH-9/2013 (H5N8)
 A/duck/Eastern China/S1109/2014 (H5N8)
 A/Hubei/29578/2016 (H5N6)
99
 A/Whooper swan/Mongolia/25/2020 (H5N6)
 A/Guangdong/18SF020/2018 (H5N6)
 A/duck/Vietnam/LBM758/2014 (H5N6)
100
 A/Whooper Swan/Hunan/4/2016 (H5N6)
 A/Greylag goose/Hunan/1/2017 (H5N6)
92
 A/teal/Tottori/1/2016 (H5N6)
 A/duck/Hyogo/1/2016 (H5N6)
 A/Mandarin duck/Korea/K16-187-3/2016 (H5N6)
99
 A/black swan/Akita/1/2016 (H5N6)
 A/tern/South Africa/1961 (H5N3)
 A/environment/Zhenjiang/C13/2013 (H5N6)
 A/duck/Vietnam/1151/2014 (H5N6)
 A/Guangzhou/39715/2014 (H5N6)
 A/duck/Guangdong/GD01/2014 (H5N6)
 A/chicken/Vietnam/NCVD-15A59/2015 (H5N6)
99
 A/Common pheasant/Hunan/11/2015 (H5N6)
93
 A/pigeon/Sichuan/NCXN29/2014 (H5N1)
 A/muscovy duck/Vietnam/LBM631/2014 (H5N1)
75
86
 A/Sichuan/26221/2014 (H5N6)
 A/duck/Eastern China/S0131/2014 (H5N2)
 A/Anhui/1/2005 (H5N1)
99
 A/goose/Guangdong/k0103/2010 (H5N5)
 A/duck/Eastern China/008/2008 (H5N5)
 A/Northern pintail/Washington/40964/2014 (H5N2)
90
 A/gyrfalcon/Washington/41088-6/2014 (H5N8)
81
 A/mallard/Alaska/AH0088535/2016 (H5N2)
 A/crane/Kagoshima/KU1/2014 (H5N8)
94
 A/duck/England/36254/2014 (H5N8)
 A/duck/Chiba/26-372-48/2014 (H5N8)
100
 A/crane/Kagoshima/KU41/2014 (H5N8)
 A/tundra swan/Korea/H411/2014 (H5N8)
 A/Chicken/Kumamoto/1-7/2014 (H5N8)
99
100
99
98
100
99
97
98
100
100
100
70
96
100
99
91
100
100
85
0.050
Supplementary Fig. S2A. Fujimoto et al.

## Slide 3
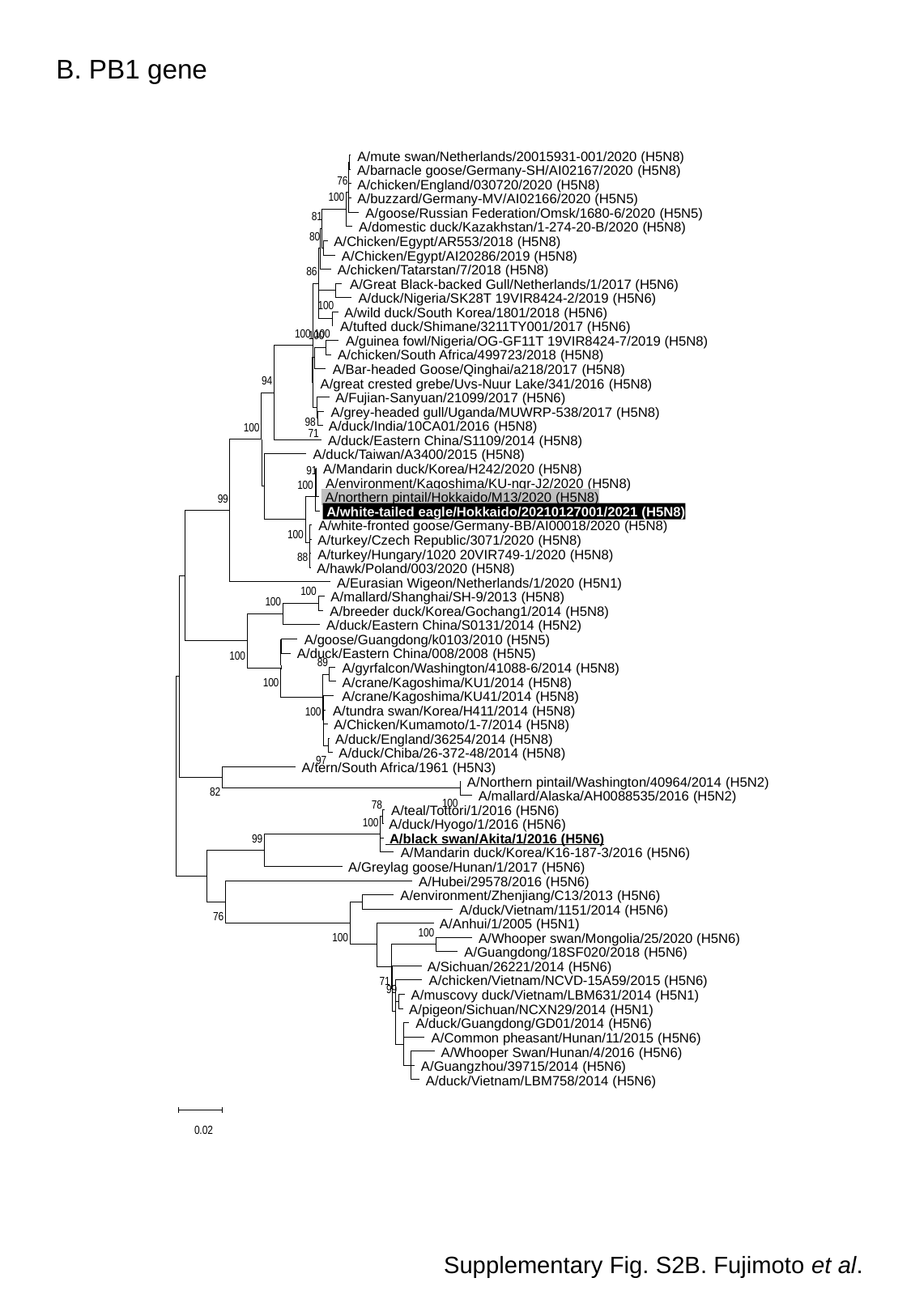

B. PB1 gene
 A/mute swan/Netherlands/20015931-001/2020 (H5N8)
 A/barnacle goose/Germany-SH/AI02167/2020 (H5N8)
 A/chicken/England/030720/2020 (H5N8)
 A/buzzard/Germany-MV/AI02166/2020 (H5N5)
 A/goose/Russian Federation/Omsk/1680-6/2020 (H5N5)
 A/domestic duck/Kazakhstan/1-274-20-B/2020 (H5N8)
 A/Chicken/Egypt/AR553/2018 (H5N8)
 A/Chicken/Egypt/AI20286/2019 (H5N8)
 A/chicken/Tatarstan/7/2018 (H5N8)
 A/Great Black-backed Gull/Netherlands/1/2017 (H5N6)
 A/duck/Nigeria/SK28T 19VIR8424-2/2019 (H5N6)
 A/wild duck/South Korea/1801/2018 (H5N6)
 A/tufted duck/Shimane/3211TY001/2017 (H5N6)
 A/guinea fowl/Nigeria/OG-GF11T 19VIR8424-7/2019 (H5N8)
 A/chicken/South Africa/499723/2018 (H5N8)
 A/Bar-headed Goose/Qinghai/a218/2017 (H5N8)
 A/great crested grebe/Uvs-Nuur Lake/341/2016 (H5N8)
 A/Fujian-Sanyuan/21099/2017 (H5N6)
 A/grey-headed gull/Uganda/MUWRP-538/2017 (H5N8)
 A/duck/India/10CA01/2016 (H5N8)
 A/duck/Eastern China/S1109/2014 (H5N8)
 A/duck/Taiwan/A3400/2015 (H5N8)
 A/Mandarin duck/Korea/H242/2020 (H5N8)
 A/environment/Kagoshima/KU-ngr-J2/2020 (H5N8)
 A/northern pintail/Hokkaido/M13/2020 (H5N8)
 A/white-tailed eagle/Hokkaido/20210127001/2021 (H5N8)
 A/white-fronted goose/Germany-BB/AI00018/2020 (H5N8)
 A/turkey/Czech Republic/3071/2020 (H5N8)
 A/turkey/Hungary/1020 20VIR749-1/2020 (H5N8)
 A/hawk/Poland/003/2020 (H5N8)
 A/Eurasian Wigeon/Netherlands/1/2020 (H5N1)
 A/mallard/Shanghai/SH-9/2013 (H5N8)
 A/breeder duck/Korea/Gochang1/2014 (H5N8)
 A/duck/Eastern China/S0131/2014 (H5N2)
 A/goose/Guangdong/k0103/2010 (H5N5)
 A/duck/Eastern China/008/2008 (H5N5)
 A/gyrfalcon/Washington/41088-6/2014 (H5N8)
 A/crane/Kagoshima/KU1/2014 (H5N8)
 A/crane/Kagoshima/KU41/2014 (H5N8)
 A/tundra swan/Korea/H411/2014 (H5N8)
 A/Chicken/Kumamoto/1-7/2014 (H5N8)
 A/duck/England/36254/2014 (H5N8)
 A/duck/Chiba/26-372-48/2014 (H5N8)
 A/tern/South Africa/1961 (H5N3)
 A/Northern pintail/Washington/40964/2014 (H5N2)
 A/mallard/Alaska/AH0088535/2016 (H5N2)
100
78
 A/teal/Tottori/1/2016 (H5N6)
100
 A/duck/Hyogo/1/2016 (H5N6)
 A/black swan/Akita/1/2016 (H5N6)
 A/Mandarin duck/Korea/K16-187-3/2016 (H5N6)
 A/Greylag goose/Hunan/1/2017 (H5N6)
 A/Hubei/29578/2016 (H5N6)
 A/environment/Zhenjiang/C13/2013 (H5N6)
 A/duck/Vietnam/1151/2014 (H5N6)
 A/Anhui/1/2005 (H5N1)
100
 A/Whooper swan/Mongolia/25/2020 (H5N6)
 A/Guangdong/18SF020/2018 (H5N6)
 A/Sichuan/26221/2014 (H5N6)
 A/chicken/Vietnam/NCVD-15A59/2015 (H5N6)
99
 A/muscovy duck/Vietnam/LBM631/2014 (H5N1)
 A/pigeon/Sichuan/NCXN29/2014 (H5N1)
 A/duck/Guangdong/GD01/2014 (H5N6)
 A/Common pheasant/Hunan/11/2015 (H5N6)
 A/Whooper Swan/Hunan/4/2016 (H5N6)
 A/Guangzhou/39715/2014 (H5N6)
 A/duck/Vietnam/LBM758/2014 (H5N6)
76
100
81
80
86
100
100
100
100
94
98
100
71
91
100
99
100
88
100
100
100
89
100
100
97
82
99
76
100
71
0.02
Supplementary Fig. S2B. Fujimoto et al.

## Slide 4
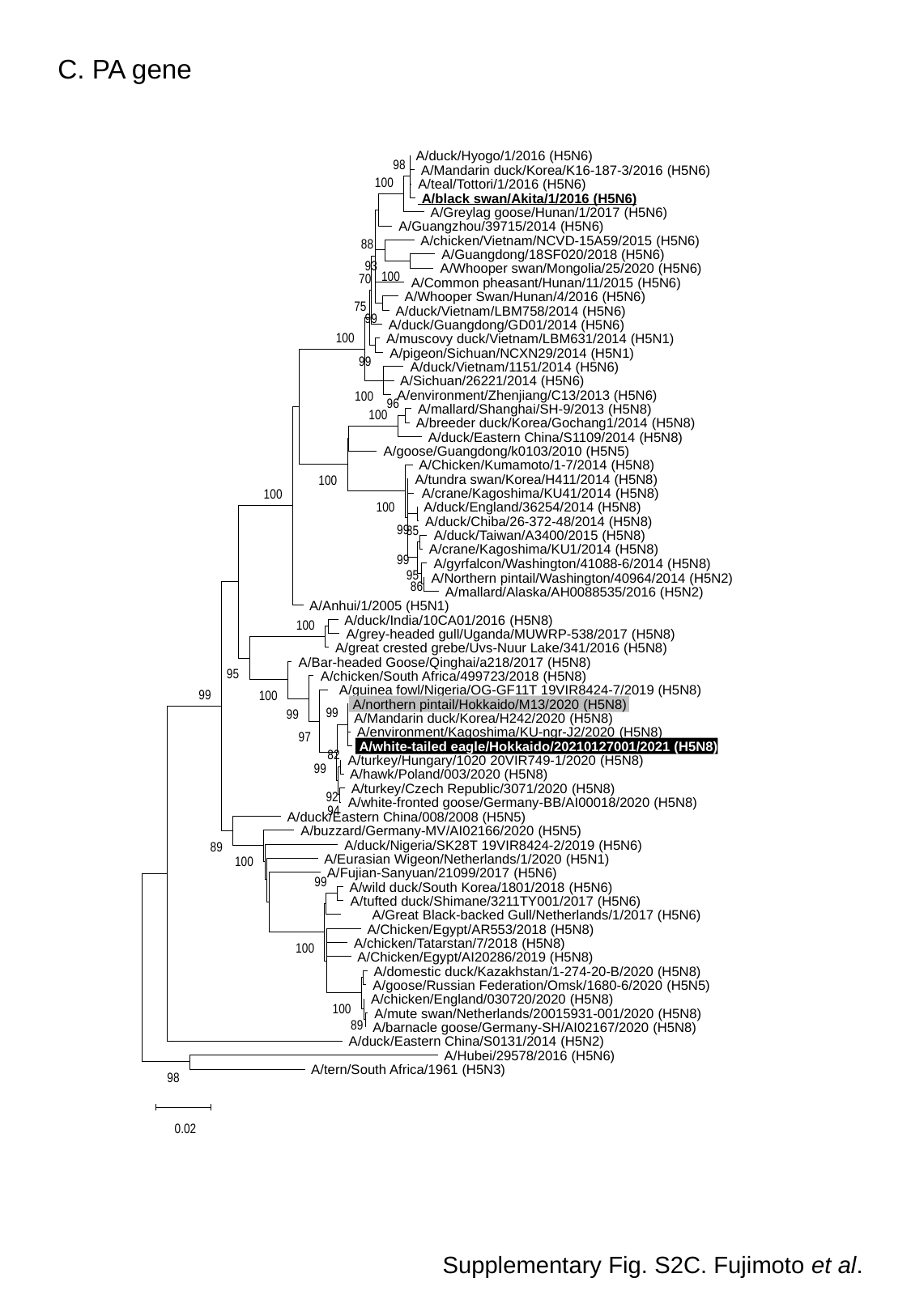

C. PA gene
 A/duck/Hyogo/1/2016 (H5N6)
 A/Mandarin duck/Korea/K16-187-3/2016 (H5N6)
 A/teal/Tottori/1/2016 (H5N6)
 A/black swan/Akita/1/2016 (H5N6)
 A/Greylag goose/Hunan/1/2017 (H5N6)
 A/Guangzhou/39715/2014 (H5N6)
 A/chicken/Vietnam/NCVD-15A59/2015 (H5N6)
 A/Guangdong/18SF020/2018 (H5N6)
 A/Whooper swan/Mongolia/25/2020 (H5N6)
 A/Common pheasant/Hunan/11/2015 (H5N6)
 A/Whooper Swan/Hunan/4/2016 (H5N6)
 A/duck/Vietnam/LBM758/2014 (H5N6)
 A/duck/Guangdong/GD01/2014 (H5N6)
 A/muscovy duck/Vietnam/LBM631/2014 (H5N1)
 A/pigeon/Sichuan/NCXN29/2014 (H5N1)
 A/duck/Vietnam/1151/2014 (H5N6)
 A/Sichuan/26221/2014 (H5N6)
 A/environment/Zhenjiang/C13/2013 (H5N6)
 A/mallard/Shanghai/SH-9/2013 (H5N8)
 A/breeder duck/Korea/Gochang1/2014 (H5N8)
 A/duck/Eastern China/S1109/2014 (H5N8)
 A/goose/Guangdong/k0103/2010 (H5N5)
 A/Chicken/Kumamoto/1-7/2014 (H5N8)
 A/tundra swan/Korea/H411/2014 (H5N8)
 A/crane/Kagoshima/KU41/2014 (H5N8)
 A/duck/England/36254/2014 (H5N8)
 A/duck/Chiba/26-372-48/2014 (H5N8)
 A/duck/Taiwan/A3400/2015 (H5N8)
 A/crane/Kagoshima/KU1/2014 (H5N8)
 A/gyrfalcon/Washington/41088-6/2014 (H5N8)
 A/Northern pintail/Washington/40964/2014 (H5N2)
 A/mallard/Alaska/AH0088535/2016 (H5N2)
 A/Anhui/1/2005 (H5N1)
 A/duck/India/10CA01/2016 (H5N8)
100
 A/grey-headed gull/Uganda/MUWRP-538/2017 (H5N8)
 A/great crested grebe/Uvs-Nuur Lake/341/2016 (H5N8)
 A/Bar-headed Goose/Qinghai/a218/2017 (H5N8)
 A/chicken/South Africa/499723/2018 (H5N8)
 A/guinea fowl/Nigeria/OG-GF11T 19VIR8424-7/2019 (H5N8)
 A/northern pintail/Hokkaido/M13/2020 (H5N8)
99
 A/Mandarin duck/Korea/H242/2020 (H5N8)
 A/environment/Kagoshima/KU-ngr-J2/2020 (H5N8)
 A/white-tailed eagle/Hokkaido/20210127001/2021 (H5N8)
 A/turkey/Hungary/1020 20VIR749-1/2020 (H5N8)
 A/hawk/Poland/003/2020 (H5N8)
 A/turkey/Czech Republic/3071/2020 (H5N8)
 A/white-fronted goose/Germany-BB/AI00018/2020 (H5N8)
94
 A/duck/Eastern China/008/2008 (H5N5)
 A/buzzard/Germany-MV/AI02166/2020 (H5N5)
 A/duck/Nigeria/SK28T 19VIR8424-2/2019 (H5N6)
 A/Eurasian Wigeon/Netherlands/1/2020 (H5N1)
 A/Fujian-Sanyuan/21099/2017 (H5N6)
99
 A/wild duck/South Korea/1801/2018 (H5N6)
 A/tufted duck/Shimane/3211TY001/2017 (H5N6)
 A/Great Black-backed Gull/Netherlands/1/2017 (H5N6)
 A/Chicken/Egypt/AR553/2018 (H5N8)
 A/chicken/Tatarstan/7/2018 (H5N8)
100
 A/Chicken/Egypt/AI20286/2019 (H5N8)
 A/domestic duck/Kazakhstan/1-274-20-B/2020 (H5N8)
 A/goose/Russian Federation/Omsk/1680-6/2020 (H5N5)
 A/chicken/England/030720/2020 (H5N8)
100
 A/mute swan/Netherlands/20015931-001/2020 (H5N8)
89
 A/barnacle goose/Germany-SH/AI02167/2020 (H5N8)
 A/duck/Eastern China/S0131/2014 (H5N2)
 A/Hubei/29578/2016 (H5N6)
 A/tern/South Africa/1961 (H5N3)
98
100
88
93
100
70
75
99
100
99
100
96
100
100
100
100
99
85
99
95
86
95
99
100
99
97
82
99
92
89
100
98
0.02
Supplementary Fig. S2C. Fujimoto et al.

## Slide 5
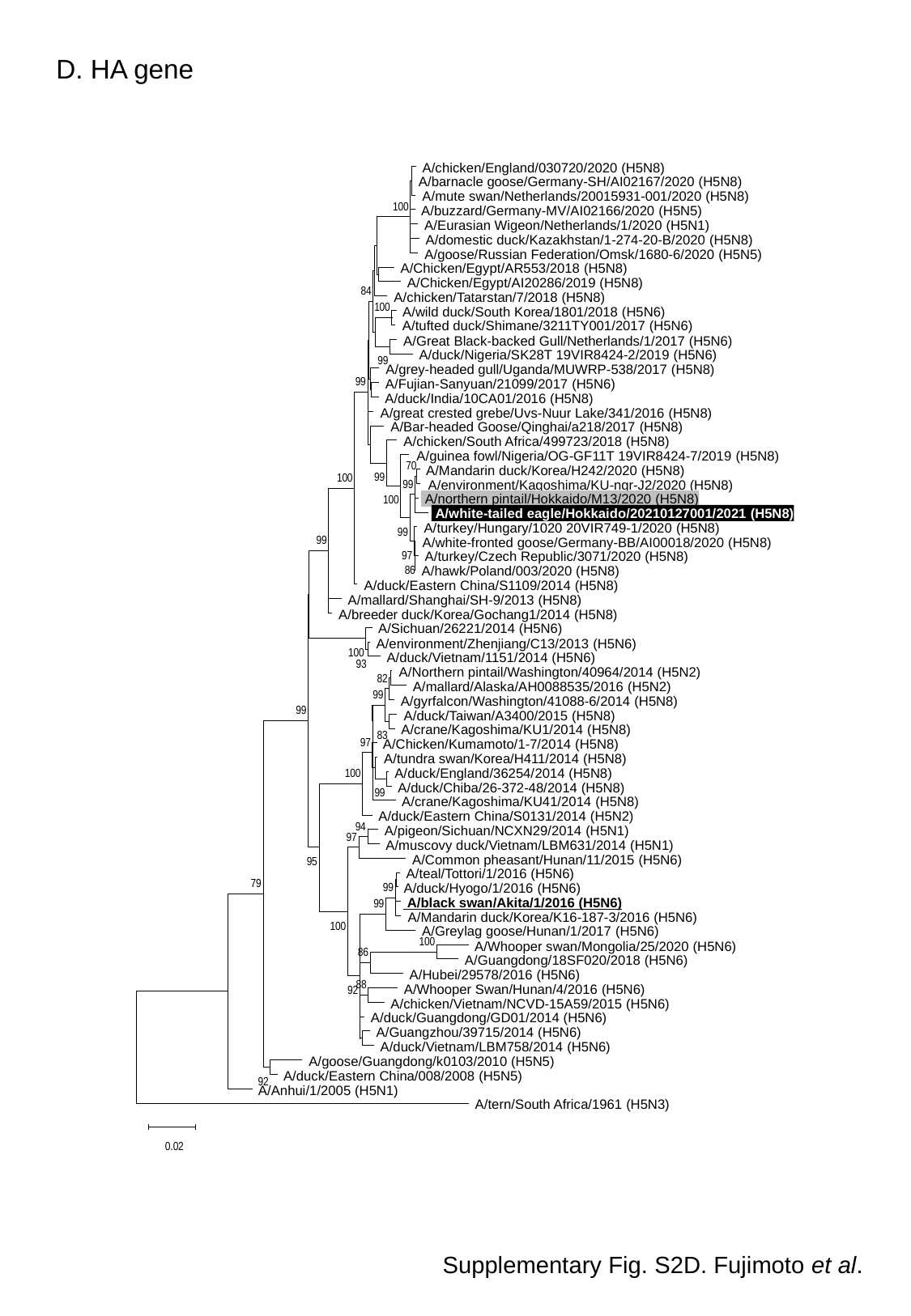

D. HA gene
 A/chicken/England/030720/2020 (H5N8)
 A/barnacle goose/Germany-SH/AI02167/2020 (H5N8)
 A/mute swan/Netherlands/20015931-001/2020 (H5N8)
 A/buzzard/Germany-MV/AI02166/2020 (H5N5)
 A/Eurasian Wigeon/Netherlands/1/2020 (H5N1)
 A/domestic duck/Kazakhstan/1-274-20-B/2020 (H5N8)
 A/goose/Russian Federation/Omsk/1680-6/2020 (H5N5)
 A/Chicken/Egypt/AR553/2018 (H5N8)
 A/Chicken/Egypt/AI20286/2019 (H5N8)
 A/chicken/Tatarstan/7/2018 (H5N8)
 A/wild duck/South Korea/1801/2018 (H5N6)
 A/tufted duck/Shimane/3211TY001/2017 (H5N6)
 A/Great Black-backed Gull/Netherlands/1/2017 (H5N6)
 A/duck/Nigeria/SK28T 19VIR8424-2/2019 (H5N6)
 A/grey-headed gull/Uganda/MUWRP-538/2017 (H5N8)
 A/Fujian-Sanyuan/21099/2017 (H5N6)
 A/duck/India/10CA01/2016 (H5N8)
 A/great crested grebe/Uvs-Nuur Lake/341/2016 (H5N8)
 A/Bar-headed Goose/Qinghai/a218/2017 (H5N8)
 A/chicken/South Africa/499723/2018 (H5N8)
 A/guinea fowl/Nigeria/OG-GF11T 19VIR8424-7/2019 (H5N8)
 A/Mandarin duck/Korea/H242/2020 (H5N8)
 A/environment/Kagoshima/KU-ngr-J2/2020 (H5N8)
 A/northern pintail/Hokkaido/M13/2020 (H5N8)
 A/white-tailed eagle/Hokkaido/20210127001/2021 (H5N8)
 A/turkey/Hungary/1020 20VIR749-1/2020 (H5N8)
 A/white-fronted goose/Germany-BB/AI00018/2020 (H5N8)
 A/turkey/Czech Republic/3071/2020 (H5N8)
 A/hawk/Poland/003/2020 (H5N8)
 A/duck/Eastern China/S1109/2014 (H5N8)
 A/mallard/Shanghai/SH-9/2013 (H5N8)
 A/breeder duck/Korea/Gochang1/2014 (H5N8)
 A/Sichuan/26221/2014 (H5N6)
 A/environment/Zhenjiang/C13/2013 (H5N6)
100
 A/duck/Vietnam/1151/2014 (H5N6)
93
 A/Northern pintail/Washington/40964/2014 (H5N2)
82
 A/mallard/Alaska/AH0088535/2016 (H5N2)
99
 A/gyrfalcon/Washington/41088-6/2014 (H5N8)
 A/duck/Taiwan/A3400/2015 (H5N8)
 A/crane/Kagoshima/KU1/2014 (H5N8)
83
 A/Chicken/Kumamoto/1-7/2014 (H5N8)
 A/tundra swan/Korea/H411/2014 (H5N8)
 A/duck/England/36254/2014 (H5N8)
 A/duck/Chiba/26-372-48/2014 (H5N8)
99
 A/crane/Kagoshima/KU41/2014 (H5N8)
 A/duck/Eastern China/S0131/2014 (H5N2)
 A/pigeon/Sichuan/NCXN29/2014 (H5N1)
 A/muscovy duck/Vietnam/LBM631/2014 (H5N1)
 A/Common pheasant/Hunan/11/2015 (H5N6)
 A/teal/Tottori/1/2016 (H5N6)
99
 A/duck/Hyogo/1/2016 (H5N6)
 A/black swan/Akita/1/2016 (H5N6)
 A/Mandarin duck/Korea/K16-187-3/2016 (H5N6)
 A/Greylag goose/Hunan/1/2017 (H5N6)
100
 A/Whooper swan/Mongolia/25/2020 (H5N6)
 A/Guangdong/18SF020/2018 (H5N6)
 A/Hubei/29578/2016 (H5N6)
 A/Whooper Swan/Hunan/4/2016 (H5N6)
 A/chicken/Vietnam/NCVD-15A59/2015 (H5N6)
 A/duck/Guangdong/GD01/2014 (H5N6)
 A/Guangzhou/39715/2014 (H5N6)
 A/duck/Vietnam/LBM758/2014 (H5N6)
 A/goose/Guangdong/k0103/2010 (H5N5)
 A/duck/Eastern China/008/2008 (H5N5)
 A/Anhui/1/2005 (H5N1)
 A/tern/South Africa/1961 (H5N3)
100
84
100
99
99
70
99
100
99
100
99
99
97
86
99
97
100
94
97
95
79
99
100
86
88
92
92
0.02
Supplementary Fig. S2D. Fujimoto et al.

## Slide 6
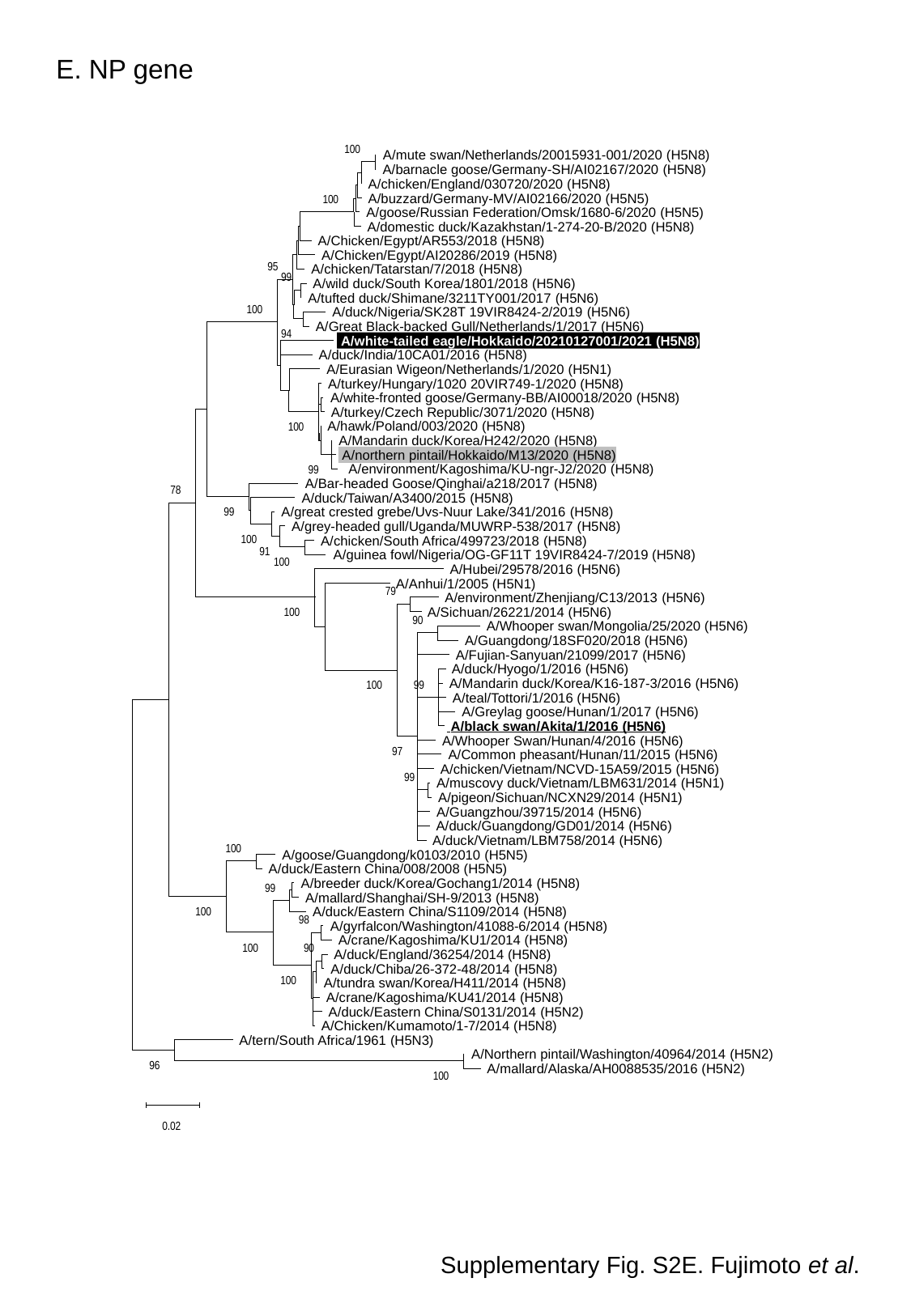

E. NP gene
 A/mute swan/Netherlands/20015931-001/2020 (H5N8)
 A/barnacle goose/Germany-SH/AI02167/2020 (H5N8)
 A/chicken/England/030720/2020 (H5N8)
 A/buzzard/Germany-MV/AI02166/2020 (H5N5)
 A/goose/Russian Federation/Omsk/1680-6/2020 (H5N5)
 A/domestic duck/Kazakhstan/1-274-20-B/2020 (H5N8)
 A/Chicken/Egypt/AR553/2018 (H5N8)
 A/Chicken/Egypt/AI20286/2019 (H5N8)
 A/chicken/Tatarstan/7/2018 (H5N8)
 A/wild duck/South Korea/1801/2018 (H5N6)
 A/tufted duck/Shimane/3211TY001/2017 (H5N6)
 A/duck/Nigeria/SK28T 19VIR8424-2/2019 (H5N6)
 A/Great Black-backed Gull/Netherlands/1/2017 (H5N6)
 A/white-tailed eagle/Hokkaido/20210127001/2021 (H5N8)
 A/duck/India/10CA01/2016 (H5N8)
 A/Eurasian Wigeon/Netherlands/1/2020 (H5N1)
 A/turkey/Hungary/1020 20VIR749-1/2020 (H5N8)
 A/white-fronted goose/Germany-BB/AI00018/2020 (H5N8)
 A/turkey/Czech Republic/3071/2020 (H5N8)
 A/hawk/Poland/003/2020 (H5N8)
 A/Mandarin duck/Korea/H242/2020 (H5N8)
 A/northern pintail/Hokkaido/M13/2020 (H5N8)
 A/environment/Kagoshima/KU-ngr-J2/2020 (H5N8)
 A/Bar-headed Goose/Qinghai/a218/2017 (H5N8)
 A/duck/Taiwan/A3400/2015 (H5N8)
 A/great crested grebe/Uvs-Nuur Lake/341/2016 (H5N8)
 A/grey-headed gull/Uganda/MUWRP-538/2017 (H5N8)
 A/chicken/South Africa/499723/2018 (H5N8)
 A/guinea fowl/Nigeria/OG-GF11T 19VIR8424-7/2019 (H5N8)
100
 A/Hubei/29578/2016 (H5N6)
 A/Anhui/1/2005 (H5N1)
79
 A/environment/Zhenjiang/C13/2013 (H5N6)
 A/Sichuan/26221/2014 (H5N6)
100
90
 A/Whooper swan/Mongolia/25/2020 (H5N6)
 A/Guangdong/18SF020/2018 (H5N6)
 A/Fujian-Sanyuan/21099/2017 (H5N6)
 A/duck/Hyogo/1/2016 (H5N6)
 A/Mandarin duck/Korea/K16-187-3/2016 (H5N6)
99
100
 A/teal/Tottori/1/2016 (H5N6)
 A/Greylag goose/Hunan/1/2017 (H5N6)
 A/black swan/Akita/1/2016 (H5N6)
 A/Whooper Swan/Hunan/4/2016 (H5N6)
97
 A/Common pheasant/Hunan/11/2015 (H5N6)
 A/chicken/Vietnam/NCVD-15A59/2015 (H5N6)
99
 A/muscovy duck/Vietnam/LBM631/2014 (H5N1)
 A/pigeon/Sichuan/NCXN29/2014 (H5N1)
 A/Guangzhou/39715/2014 (H5N6)
 A/duck/Guangdong/GD01/2014 (H5N6)
 A/duck/Vietnam/LBM758/2014 (H5N6)
100
 A/goose/Guangdong/k0103/2010 (H5N5)
 A/duck/Eastern China/008/2008 (H5N5)
 A/breeder duck/Korea/Gochang1/2014 (H5N8)
99
 A/mallard/Shanghai/SH-9/2013 (H5N8)
 A/duck/Eastern China/S1109/2014 (H5N8)
100
98
 A/gyrfalcon/Washington/41088-6/2014 (H5N8)
 A/crane/Kagoshima/KU1/2014 (H5N8)
100
90
 A/duck/England/36254/2014 (H5N8)
 A/duck/Chiba/26-372-48/2014 (H5N8)
100
 A/tundra swan/Korea/H411/2014 (H5N8)
 A/crane/Kagoshima/KU41/2014 (H5N8)
 A/duck/Eastern China/S0131/2014 (H5N2)
 A/Chicken/Kumamoto/1-7/2014 (H5N8)
 A/tern/South Africa/1961 (H5N3)
 A/Northern pintail/Washington/40964/2014 (H5N2)
 A/mallard/Alaska/AH0088535/2016 (H5N2)
100
100
100
95
99
100
94
100
99
78
99
100
91
96
0.02
Supplementary Fig. S2E. Fujimoto et al.

## Slide 7
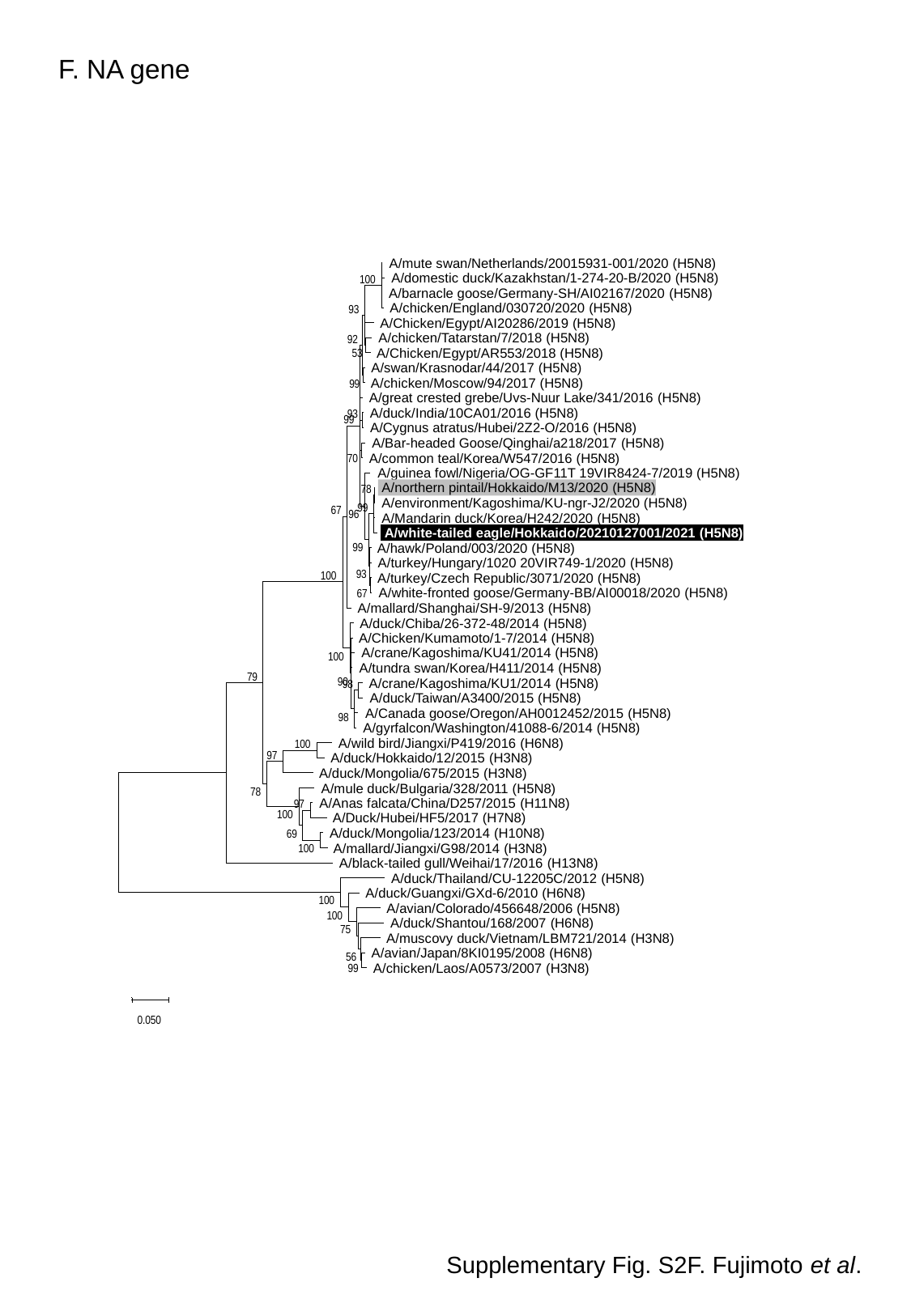

F. NA gene
 A/mute swan/Netherlands/20015931-001/2020 (H5N8)
 A/domestic duck/Kazakhstan/1-274-20-B/2020 (H5N8)
100
 A/barnacle goose/Germany-SH/AI02167/2020 (H5N8)
 A/chicken/England/030720/2020 (H5N8)
93
 A/Chicken/Egypt/AI20286/2019 (H5N8)
 A/chicken/Tatarstan/7/2018 (H5N8)
92
 A/Chicken/Egypt/AR553/2018 (H5N8)
53
 A/swan/Krasnodar/44/2017 (H5N8)
 A/chicken/Moscow/94/2017 (H5N8)
99
 A/great crested grebe/Uvs-Nuur Lake/341/2016 (H5N8)
 A/duck/India/10CA01/2016 (H5N8)
93
99
 A/Cygnus atratus/Hubei/2Z2-O/2016 (H5N8)
 A/Bar-headed Goose/Qinghai/a218/2017 (H5N8)
 A/common teal/Korea/W547/2016 (H5N8)
70
 A/guinea fowl/Nigeria/OG-GF11T 19VIR8424-7/2019 (H5N8)
 A/northern pintail/Hokkaido/M13/2020 (H5N8)
78
 A/environment/Kagoshima/KU-ngr-J2/2020 (H5N8)
99
67
96
 A/Mandarin duck/Korea/H242/2020 (H5N8)
 A/white-tailed eagle/Hokkaido/20210127001/2021 (H5N8)
 A/hawk/Poland/003/2020 (H5N8)
99
 A/turkey/Hungary/1020 20VIR749-1/2020 (H5N8)
93
100
 A/turkey/Czech Republic/3071/2020 (H5N8)
 A/white-fronted goose/Germany-BB/AI00018/2020 (H5N8)
67
 A/mallard/Shanghai/SH-9/2013 (H5N8)
 A/duck/Chiba/26-372-48/2014 (H5N8)
 A/Chicken/Kumamoto/1-7/2014 (H5N8)
 A/crane/Kagoshima/KU41/2014 (H5N8)
100
 A/tundra swan/Korea/H411/2014 (H5N8)
79
90
 A/crane/Kagoshima/KU1/2014 (H5N8)
98
 A/duck/Taiwan/A3400/2015 (H5N8)
 A/Canada goose/Oregon/AH0012452/2015 (H5N8)
98
 A/gyrfalcon/Washington/41088-6/2014 (H5N8)
 A/wild bird/Jiangxi/P419/2016 (H6N8)
100
97
 A/duck/Hokkaido/12/2015 (H3N8)
 A/duck/Mongolia/675/2015 (H3N8)
 A/mule duck/Bulgaria/328/2011 (H5N8)
78
 A/Anas falcata/China/D257/2015 (H11N8)
97
100
 A/Duck/Hubei/HF5/2017 (H7N8)
 A/duck/Mongolia/123/2014 (H10N8)
69
 A/mallard/Jiangxi/G98/2014 (H3N8)
100
 A/black-tailed gull/Weihai/17/2016 (H13N8)
 A/duck/Thailand/CU-12205C/2012 (H5N8)
 A/duck/Guangxi/GXd-6/2010 (H6N8)
100
 A/avian/Colorado/456648/2006 (H5N8)
100
 A/duck/Shantou/168/2007 (H6N8)
75
 A/muscovy duck/Vietnam/LBM721/2014 (H3N8)
 A/avian/Japan/8KI0195/2008 (H6N8)
56
 A/chicken/Laos/A0573/2007 (H3N8)
99
0.050
Supplementary Fig. S2F. Fujimoto et al.

## Slide 8
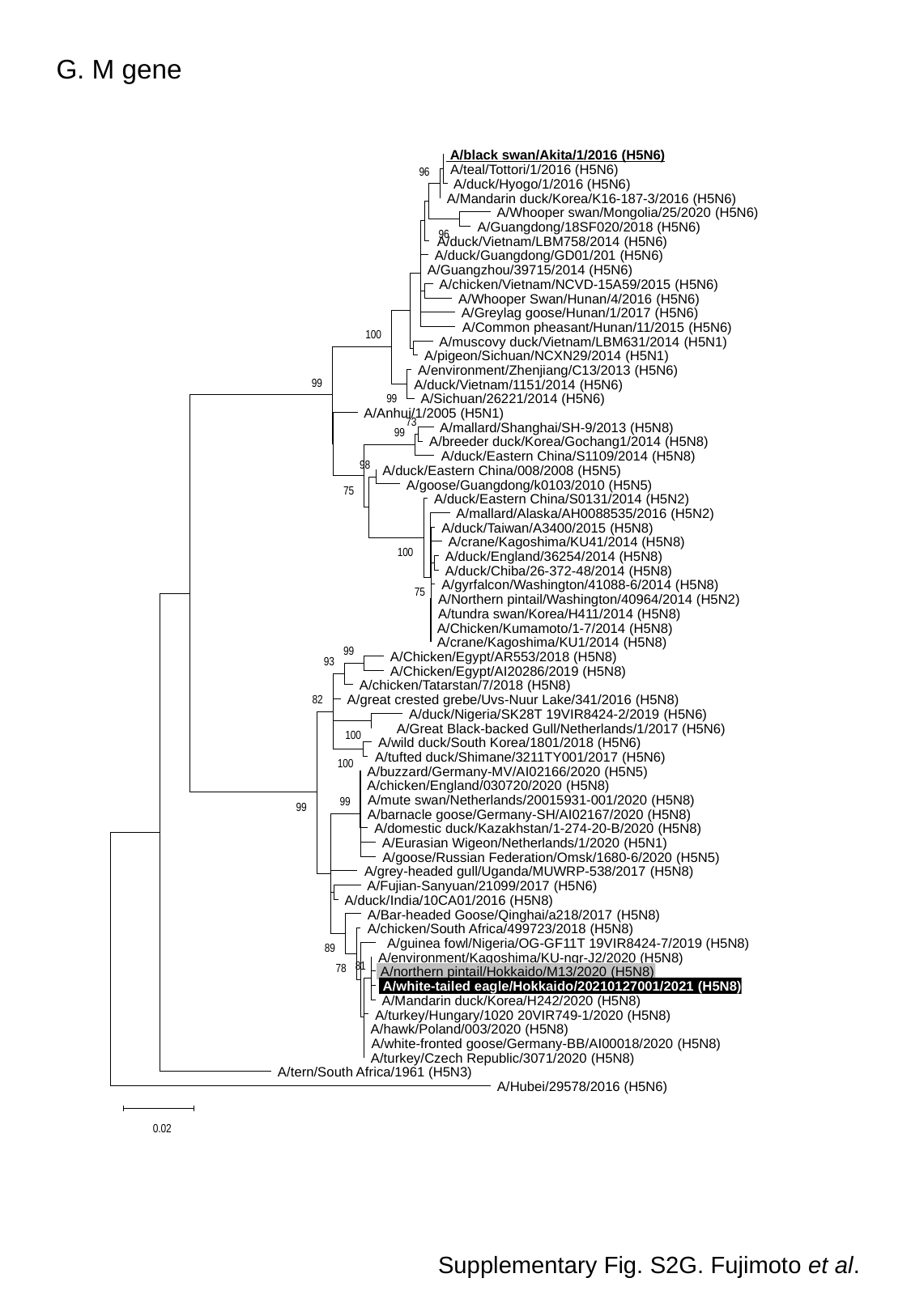

G. M gene
 A/black swan/Akita/1/2016 (H5N6)
 A/teal/Tottori/1/2016 (H5N6)
96
 A/duck/Hyogo/1/2016 (H5N6)
 A/Mandarin duck/Korea/K16-187-3/2016 (H5N6)
 A/Whooper swan/Mongolia/25/2020 (H5N6)
 A/Guangdong/18SF020/2018 (H5N6)
96
 A/duck/Vietnam/LBM758/2014 (H5N6)
 A/duck/Guangdong/GD01/201 (H5N6)
 A/Guangzhou/39715/2014 (H5N6)
 A/chicken/Vietnam/NCVD-15A59/2015 (H5N6)
 A/Whooper Swan/Hunan/4/2016 (H5N6)
 A/Greylag goose/Hunan/1/2017 (H5N6)
 A/Common pheasant/Hunan/11/2015 (H5N6)
100
 A/muscovy duck/Vietnam/LBM631/2014 (H5N1)
 A/pigeon/Sichuan/NCXN29/2014 (H5N1)
 A/environment/Zhenjiang/C13/2013 (H5N6)
99
 A/duck/Vietnam/1151/2014 (H5N6)
 A/Sichuan/26221/2014 (H5N6)
99
 A/Anhui/1/2005 (H5N1)
73
 A/mallard/Shanghai/SH-9/2013 (H5N8)
99
 A/breeder duck/Korea/Gochang1/2014 (H5N8)
 A/duck/Eastern China/S1109/2014 (H5N8)
98
 A/duck/Eastern China/008/2008 (H5N5)
 A/goose/Guangdong/k0103/2010 (H5N5)
75
 A/duck/Eastern China/S0131/2014 (H5N2)
 A/mallard/Alaska/AH0088535/2016 (H5N2)
 A/duck/Taiwan/A3400/2015 (H5N8)
 A/crane/Kagoshima/KU41/2014 (H5N8)
100
 A/duck/England/36254/2014 (H5N8)
 A/duck/Chiba/26-372-48/2014 (H5N8)
 A/gyrfalcon/Washington/41088-6/2014 (H5N8)
75
 A/Northern pintail/Washington/40964/2014 (H5N2)
 A/tundra swan/Korea/H411/2014 (H5N8)
 A/Chicken/Kumamoto/1-7/2014 (H5N8)
 A/crane/Kagoshima/KU1/2014 (H5N8)
99
 A/Chicken/Egypt/AR553/2018 (H5N8)
93
 A/Chicken/Egypt/AI20286/2019 (H5N8)
 A/chicken/Tatarstan/7/2018 (H5N8)
 A/great crested grebe/Uvs-Nuur Lake/341/2016 (H5N8)
82
 A/duck/Nigeria/SK28T 19VIR8424-2/2019 (H5N6)
 A/Great Black-backed Gull/Netherlands/1/2017 (H5N6)
100
 A/wild duck/South Korea/1801/2018 (H5N6)
 A/tufted duck/Shimane/3211TY001/2017 (H5N6)
100
 A/buzzard/Germany-MV/AI02166/2020 (H5N5)
 A/chicken/England/030720/2020 (H5N8)
 A/mute swan/Netherlands/20015931-001/2020 (H5N8)
99
99
 A/barnacle goose/Germany-SH/AI02167/2020 (H5N8)
 A/domestic duck/Kazakhstan/1-274-20-B/2020 (H5N8)
 A/Eurasian Wigeon/Netherlands/1/2020 (H5N1)
 A/goose/Russian Federation/Omsk/1680-6/2020 (H5N5)
 A/grey-headed gull/Uganda/MUWRP-538/2017 (H5N8)
 A/Fujian-Sanyuan/21099/2017 (H5N6)
 A/duck/India/10CA01/2016 (H5N8)
 A/Bar-headed Goose/Qinghai/a218/2017 (H5N8)
 A/chicken/South Africa/499723/2018 (H5N8)
 A/guinea fowl/Nigeria/OG-GF11T 19VIR8424-7/2019 (H5N8)
89
 A/environment/Kagoshima/KU-ngr-J2/2020 (H5N8)
81
78
 A/northern pintail/Hokkaido/M13/2020 (H5N8)
 A/white-tailed eagle/Hokkaido/20210127001/2021 (H5N8)
 A/Mandarin duck/Korea/H242/2020 (H5N8)
 A/turkey/Hungary/1020 20VIR749-1/2020 (H5N8)
 A/hawk/Poland/003/2020 (H5N8)
 A/white-fronted goose/Germany-BB/AI00018/2020 (H5N8)
 A/turkey/Czech Republic/3071/2020 (H5N8)
 A/tern/South Africa/1961 (H5N3)
 A/Hubei/29578/2016 (H5N6)
0.02
Supplementary Fig. S2G. Fujimoto et al.

## Slide 9
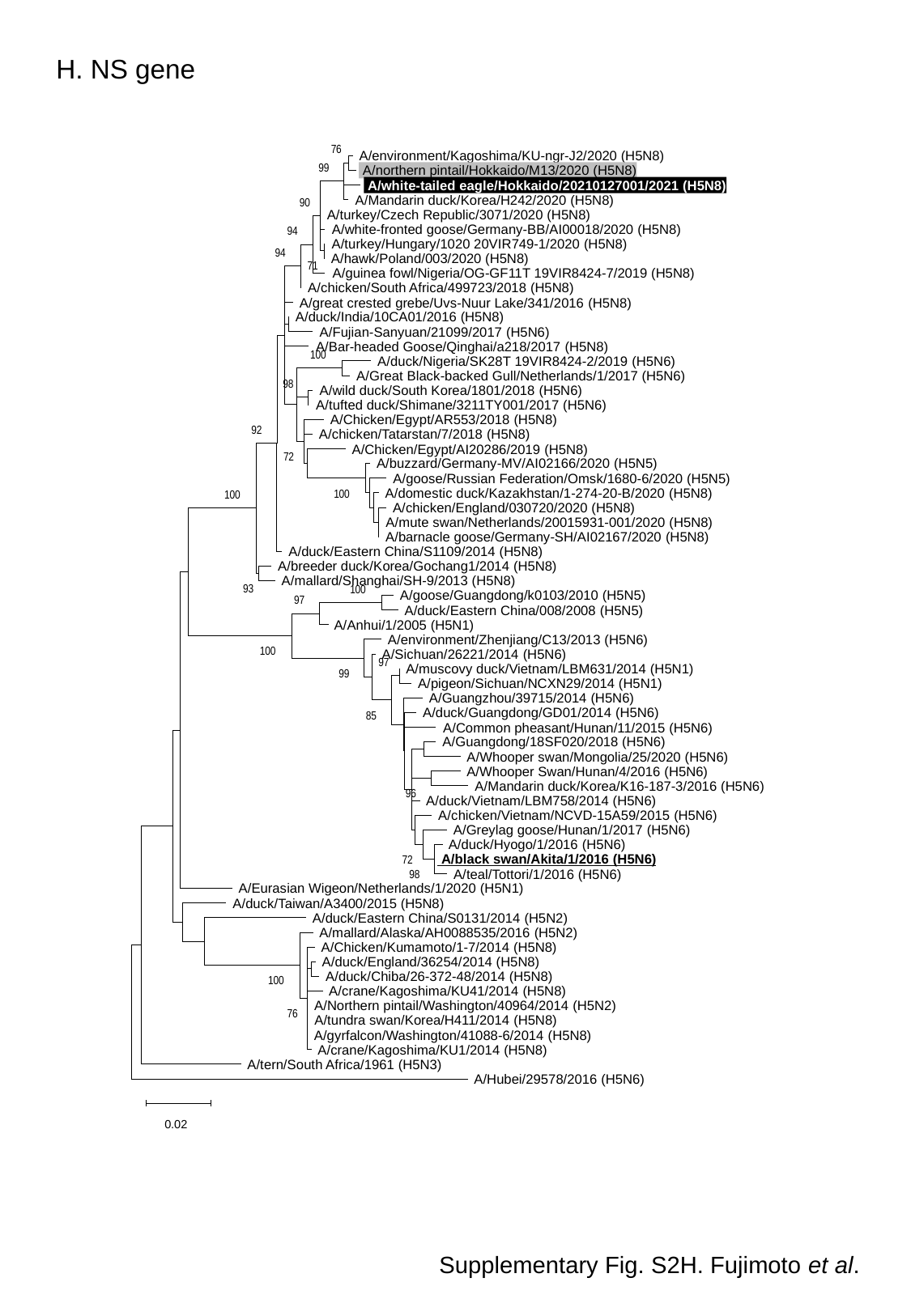

H. NS gene
76
 A/environment/Kagoshima/KU-ngr-J2/2020 (H5N8)
99
 A/northern pintail/Hokkaido/M13/2020 (H5N8)
 A/white-tailed eagle/Hokkaido/20210127001/2021 (H5N8)
 A/Mandarin duck/Korea/H242/2020 (H5N8)
 A/turkey/Czech Republic/3071/2020 (H5N8)
 A/white-fronted goose/Germany-BB/AI00018/2020 (H5N8)
 A/turkey/Hungary/1020 20VIR749-1/2020 (H5N8)
 A/hawk/Poland/003/2020 (H5N8)
 A/guinea fowl/Nigeria/OG-GF11T 19VIR8424-7/2019 (H5N8)
 A/chicken/South Africa/499723/2018 (H5N8)
 A/great crested grebe/Uvs-Nuur Lake/341/2016 (H5N8)
 A/duck/India/10CA01/2016 (H5N8)
 A/Fujian-Sanyuan/21099/2017 (H5N6)
 A/Bar-headed Goose/Qinghai/a218/2017 (H5N8)
100
 A/duck/Nigeria/SK28T 19VIR8424-2/2019 (H5N6)
 A/Great Black-backed Gull/Netherlands/1/2017 (H5N6)
 A/wild duck/South Korea/1801/2018 (H5N6)
 A/tufted duck/Shimane/3211TY001/2017 (H5N6)
 A/Chicken/Egypt/AR553/2018 (H5N8)
 A/chicken/Tatarstan/7/2018 (H5N8)
 A/Chicken/Egypt/AI20286/2019 (H5N8)
 A/buzzard/Germany-MV/AI02166/2020 (H5N5)
 A/goose/Russian Federation/Omsk/1680-6/2020 (H5N5)
 A/domestic duck/Kazakhstan/1-274-20-B/2020 (H5N8)
100
 A/chicken/England/030720/2020 (H5N8)
 A/mute swan/Netherlands/20015931-001/2020 (H5N8)
 A/barnacle goose/Germany-SH/AI02167/2020 (H5N8)
 A/duck/Eastern China/S1109/2014 (H5N8)
 A/breeder duck/Korea/Gochang1/2014 (H5N8)
 A/mallard/Shanghai/SH-9/2013 (H5N8)
93
100
 A/goose/Guangdong/k0103/2010 (H5N5)
97
 A/duck/Eastern China/008/2008 (H5N5)
 A/Anhui/1/2005 (H5N1)
 A/environment/Zhenjiang/C13/2013 (H5N6)
100
 A/Sichuan/26221/2014 (H5N6)
97
 A/muscovy duck/Vietnam/LBM631/2014 (H5N1)
99
 A/pigeon/Sichuan/NCXN29/2014 (H5N1)
 A/Guangzhou/39715/2014 (H5N6)
 A/duck/Guangdong/GD01/2014 (H5N6)
85
 A/Common pheasant/Hunan/11/2015 (H5N6)
 A/Guangdong/18SF020/2018 (H5N6)
 A/Whooper swan/Mongolia/25/2020 (H5N6)
 A/Whooper Swan/Hunan/4/2016 (H5N6)
 A/Mandarin duck/Korea/K16-187-3/2016 (H5N6)
96
 A/duck/Vietnam/LBM758/2014 (H5N6)
 A/chicken/Vietnam/NCVD-15A59/2015 (H5N6)
 A/Greylag goose/Hunan/1/2017 (H5N6)
 A/duck/Hyogo/1/2016 (H5N6)
 A/black swan/Akita/1/2016 (H5N6)
72
 A/teal/Tottori/1/2016 (H5N6)
98
 A/Eurasian Wigeon/Netherlands/1/2020 (H5N1)
 A/duck/Taiwan/A3400/2015 (H5N8)
 A/duck/Eastern China/S0131/2014 (H5N2)
 A/mallard/Alaska/AH0088535/2016 (H5N2)
 A/Chicken/Kumamoto/1-7/2014 (H5N8)
 A/duck/England/36254/2014 (H5N8)
 A/duck/Chiba/26-372-48/2014 (H5N8)
100
 A/crane/Kagoshima/KU41/2014 (H5N8)
 A/Northern pintail/Washington/40964/2014 (H5N2)
76
 A/tundra swan/Korea/H411/2014 (H5N8)
 A/gyrfalcon/Washington/41088-6/2014 (H5N8)
 A/crane/Kagoshima/KU1/2014 (H5N8)
 A/tern/South Africa/1961 (H5N3)
 A/Hubei/29578/2016 (H5N6)
90
94
94
71
98
92
72
100
0.02
Supplementary Fig. S2H. Fujimoto et al.
