# Supplementary material for: Experimental and natural infections of white-tailed sea eagles (Haliaeetus albicilla) with high pathogenicity avian influenza virus of H5 subtype
Source: Front Microbiol. 2022 Oct 3;13:1007350. doi: 10.3389/fmicb.2022.1007350 (PMC9574225; doi:10.3389/fmicb.2022.1007350)
Supplement: Supplementary file 2 [file Data_Sheet_1.pdf]

Supplementary Figure S1. Changes in body temperature and body weight of white-tailed sea eagles experimentally infected with H5N6 HPAI virus. Body temperature (A) and body weight (B) of the two inoculated eagles (birds A and B) and of the contact eagle (bird C) that was housed adjacent to bird B were measured using a rectal thermometer every morning.

Supplementary Figure S2. Phylogenetic trees based on all eight-segmented genes. The nucleotide sequences of the PB2 (A), PB1 (B), PA (C), HA (D), NP (E), NA (F), M (G), and NS (H) genes from A/white-tailed sea eagle/Hokkaido/20210127001/2021 (H5N8) (highlighted in black), A/black swan/Akita/1/2016 (H5N6) (underlined), and A/northern pintail/Hokkaido/M13/2020 (H5N8) (highlighted in gray) were phylogenetically analyzed and compared with their counterparts from the representative viruses used in a previous study (Isoda et al., 2020) (A, B, C, D, E, G, and H) and from representative N8 low pathogenicity avian influenza viruses (F). The phylogenetic trees were constructed using the maximum likelihood method with 1,000 bootstrap replicates. Bootstrap values of >70% are shown at the nodes. The scale bar indicates the number of nucleotide substitutions per site.

Isoda, N., Twabela, A. T., Bazarragchaa, E., Ogasawara, K., Hayashi, H., Wang, Z. J., et al. (2020) Re-invasion of H5N8 high pathogenicity avian influenza virus clade 2.3.4.4b in Hokkaido, Japan, 2020. *Viruses* 12. doi: 10.3390/v12121439.
